# Antiplasmodial Compounds from Deep-Water Marine Invertebrates

**DOI:** 10.3390/md19040179

**Published:** 2021-03-25

**Authors:** Amy E. Wright, Jennifer E. Collins, Bracken Roberts, Jill C. Roberts, Priscilla L. Winder, John K. Reed, Maria Cristina Diaz, Shirley A. Pomponi, Debopam Chakrabarti

**Affiliations:** 1 Harbor Branch Oceanographic Institute, Florida Atlantic University, 5600 US Highway 1 North, Fort Pierce, FL 34946, USA; jrober90@fau.edu (J.C.R.); PWINDER@fau.edu (P.L.W.); Jreed12@fau.edu (J.K.R.); taxochica@gmail.com (M.C.D.); SPomponi@fau.edu (S.A.P.); 2 Burnett School of Biomedical Sciences, University of Central Florida, Orlando, FL 32826, USA; JLeadem@knights.ucf.edu (J.E.C.); Bracken.Roberts@knights.ucf.edu (B.R.)

**Keywords:** bebrycin A, nitenin, antiplasmodial, *Bebryce grandis*, *Spongia lamella*, marine natural product, malaria, *P. falciparum*

## Abstract

Novel drug leads for malaria therapy are urgently needed because of the widespread emergence of resistance to all available drugs. Screening of the Harbor Branch enriched fraction library against the *Plasmodium falciparum* chloroquine-resistant strain (Dd2) followed by bioassay-guided fractionation led to the identification of two potent antiplasmodials; a novel diterpene designated as bebrycin A (**1**) and the known C21 degraded terpene nitenin (**2**). A SYBR Green I assay was used to establish a Dd2 EC_50_ of 1.08 ± 0.21 and 0.29 ± 0.02 µM for bebrycin A and nitenin, respectively. Further analysis was then performed to assess the stage specificity of the inhibitors antiplasmodial effects on the Dd2 intraerythrocytic life cycle. Exposure to bebrycin A was found to block parasite maturation at the schizont stage if added any time prior to late schizogony at 42 hours post invasion, (HPI). In contrast, early life cycle exposure to nitenin (prior to 18 HPI) was identified as crucial to parasite inhibition, suggesting nitenin may target the maturation of the parasite during the transition from ring to early trophozoite (6–18 HPI), a novel property among known antimalarials.

## 1. Introduction

Worldwide, malaria continues to be a prevalent infectious disease with an estimated 209 million clinical cases in 2019, with children and pregnant women being most at risk [1,2]. There remain limited treatment options due to the widespread prevalence of drug resistance among the disease causing *Plasmodium spp.* parasite. Even artemisinin-based combination therapies (ACTs), which are the front-line therapeutic options for uncomplicated *Plasmodium falciparum* malaria, are showing signs of ineffectiveness in a wide area of Southeast Asia due to point mutations in *kelch13* [3,4,5]. In addition, parasites are exhibiting signs of resistance against artemisinin partner drugs [6,7]. This grim situation underscores the urgent need to develop novel antimalarials acting on targets different from existing therapeutics. Marine organisms have long been a source of novel natural products with unique chemical scaffolds possessing a variety of potent biological activities [8]. This rich marine biodiversity provides us an enormous opportunity to identify novel antimalarial leads from specialized metabolites of marine organisms [9].

**Figure 1:**
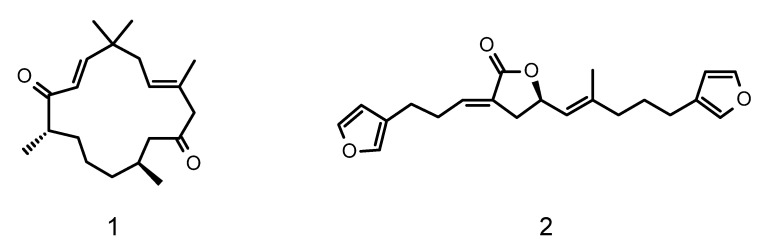
Structures of bebrycin A (**1**) and nitenin (**2**).

The Harbor Branch Oceanographic Institute of Florida Atlantic University has a substantial collection of marine invertebrates, many collected in deep-water habitats using manned submersibles. A sub-set of chemically rich organisms in the collection has been fractionated using medium pressure liquid chromatography to create a library of enriched fractions [10]. Screening of this library for antiplasmodial activity led to the identification of 165 fractions from 85 taxonomically distinct organisms that inhibit the proliferation of the parasite at concentrations of ≤5 µg/mL [11]. This manuscript describes the isolation, structure elucidation, and biological activity of two classes of compounds identified from this screening effort. They include a novel diterpene which we have designated as bebrycin A (**1**) from the octocoral *Bebryce grandis* [12], and the previously reported C-21 degraded terpenoid nitenin (**2**) [13,14] from a specimen of *Spongia lamella* (Figure 1). Fractions from both organisms showed substantial activity against the Dd2 chloroquine resistant strain of *P. falciparum* and were selected for further fractionation and structure elucidation.

## 2. Results

### 2.1. Chemical Analysis

The sample of *Bebryce grandis* [Phylum: Cnidaria, Class: Anthozoa, Subclass: Octocorallia, Order: Alcyonacea, Family: Plexauridae] from which bebrycin A (**1**) was isolated was collected using the *Johnson-Sea-Link* II submersible at a depth of 131 m off Ocean Cay, Bahamas, approximately 20 nautical miles south of Bimini. The sample was frozen at −20 °C immediately after collection and stored frozen until work-up. The frozen octocoral was extracted exhaustively with ethanol (EtOH). After concentration by distillation under reduced pressure, the extract was partitioned between *n*-butanol and H_2_O. The *n*-butanol partition was fractionated by medium pressure liquid chromatography (MPLC) using a Combi-*Flash R*_f_4x and a Redi-Sep Gold C-18 column eluted with a linear gradient of CH_3_CN and H_2_O. Final purification was achieved using semi-preparative HPLC on a Vydac C-18 column with isocratic elution (CH_3_CN:H_2_O (55:45 *v/v*)) to yield **1** (1.9 mg, 1.75 × 10^−3^% of wet weight) as an amorphous white solid. Two additional specimens were also found to contain **1** and were used to isolate additional material for bioassays.

**Figure 2:**
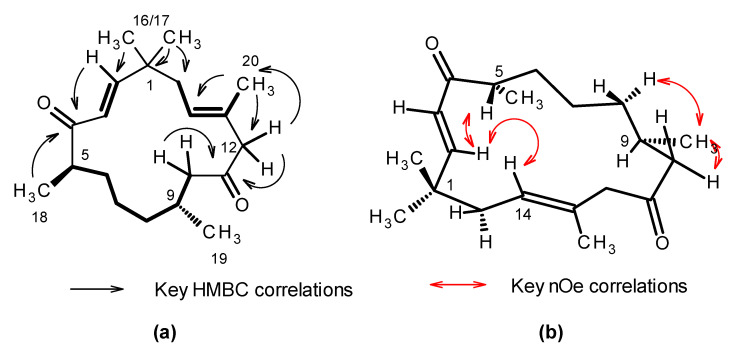
Key NMR data for **1**. (**a**) Bolded bonds indicate spin systems defined for **1** from interpretation of the 2D-DQF COSY spectra. Black arrows indicate key ^1^H-^13^C long-range couplings observed in the 2D-*g*HMBC spectrum that tie the spin systems together. (**b**) Red arrows indicate key nuclear Overhauser enhancements (nOe) observed in the 2D-NOESY and 1D-dpfgse-noe spectra.

Inspection of the ^13^C NMR data Table 1 coupled with high resolution electrospray ionization mass spectrometry (HR-ESIMS) analysis of **1** suggested a molecular formula of C_20_H_32_O_2_ requiring 5 degrees of unsaturation (Figures S5 and S23). A strong absorption observed at 1705 cm^−1^ in the IR spectrum suggested the presence of conjugated ketone functionality in the molecule. The ^13^C NMR spectra observed in *d_4_*-methanol showed the presence of two resonances attributable to ketones (δ_C_ 211.8 and 206.7); three olefinic methine carbons (δ_C_ 157.3, 126.9 and 126.5); and one quaternary olefinic carbon (δ_C_ 132.8). No additional unsaturation was apparent from the NMR spectra and therefore the structure of **1** was assigned one ring. The ^1^H NMR data Table 1 and edited *g*-HSQC (Figures S4, S6 and S7) confirmed the presence of the two double bonds, as well as identified two sp^3^ hybridized methine carbons, six methylene groups, and five methyl groups in **1**. Interpretation of the 2D g-DQF COSY spectrum (Figures S8–S10) allowed for the assignment of three spin systems in **1** (bolded bonds in Figure 2) along with an isolated methylene group appearing as an AX pattern (δ_H_ 3.00 d (*J* = 12.4 Hz) and 2.92 d (*J* = 12.4 Hz)). Data from the 2D-gHMBC experiment (Figures S11–S15) allowed the spin systems to be tied together as follows (key correlations in the HMBC spectrum are shown in Figure 2). The singlet methyl groups observed at δ_H_ 1.12 and 1.08 (H_3_-16 and H_3_-17) both have strong correlations to the quaternary carbon observed at δ_C_ 39.1 (C-1), the methylene carbon observed at δ_C_ 41.7 (C-15) and the olefinic carbon observed at δ_C_ 157.3 (C-2). They also showed strong correlations to each other δ_H_ 1.12 (H_3_-16) to δ_C_ 26.6 (C-17) and δ_H_ 1.08 (H_3_-17) to δ_C_ 27.4 (C-16), suggesting the presence of geminal methyl groups attached to a quaternary carbon that connects the C-2/C-3 olefin spin system with the C-15 to C-20 spin system. Correlations observed in the 2D-gHMBC spectrum between H-2 and C-1, C-15 and C-16, as well as correlations from H-15ab to C-1, C-2, C-16, and C-17, further support this assignment. The observed chemical shifts for H-2 (δ_H_ 6.83) and H-3 (δ_H_ 6.10), along with long range couplings observed in the g-HMBC spectrum for both H-2 and H-3 to a ketone resonance observed at δ_C_ 206.7 (C-4), allow for incorporation of the first ketone moiety as C-4. This was further extended to incorporate the C-5 to C-10 spin system based upon ^1^H-^13^C long range couplings observed in the g-HMBC spectrum from H_3_-18 (δ_H_ 1.01) to the C-4 ketone resonance. Correlations from H-10ab (δ_H_ 2.59 and 2.05) to the second ketone carbon observed at δ_C_ 211.8 allowed for placement of the final ketone as C-11. Both protons of an isolated methylene group observed at δ_H_ 3.00 and 2.92 showed strong correlations in the HMBC spectrum to both the C-11 ketone carbon and to the C-10 methylene group, allowing for its assignment as C-12. These protons also showed correlations in the HMBC spectrum to the olefinic carbon C-13 (δ_C_ 132.8), as well as the olefinic methyl C-20 (δ_C_ 17.3), allowing for the final connection and closing of the macrocyclic ring to form a 15 carbon macrocyclic structure.

The geometry of the C-2–C-3 double bond was assigned to be *E*-configuration based upon the large coupling constant between H-2 and H-3 (*J* = 15.8 Hz). The C-13–C-14 double bond was assigned as *E* configuration due to the observation of an nOe between H_3_-20 and H-15ab, and between H-14 and H-12ab. Additional support for the *E* configuration is the chemical shift of C-20 (δ_C_ 17.3). Methyl groups on *E*-configured trisubstituted olefins are observed below 20 ppm [15,16]. Assignment of the relative configuration of the molecule is complicated by the flexibility of the 15-membered ring. C-5 is tentatively assigned as S* based upon the following data: strong nOes are observed between H-5 and both H-2 and H-3 in both the 2D-NOESY and 1D-dpfgse nOe spectra (Figure 2 and Figures S16–S22), suggesting that this proton faces towards the center of the macrocycle. H-2 shows a strong nOe to the olefinic proton H-14 and to the H_2-_15 methylene protons, suggesting that these atoms also face towards the center of the macrocyclic ring. The configuration at C-9 has been tentatively assigned as R*, but due to conformational flexibility of the 15 membered ring the data to support this is limited. H-9 appears as an 8 line multiplet consistent with coupling to seven protons, all with similar J couplings of 6 to 7 Hz. In the 2D-NOESY spectrum H_3_-19 has correlations to H-8a, H-10a and H-10b. These nOes suggest that H_3_-19 is in a pseudo-equatorial position. The 15 ring macrocycle has substantial conformational flexibility and the assignments are considered tentative. The absolute configuration has not been assigned.

Nitenin (**2**) was isolated from a sample of *Spongia lamella* [Phylum: Porifera, Class: Demospongiae, Order: Dictyoceratida, Family: Spongiidae] collected using the *Johnson-Sea-Link* I submersible, from a rock outcrop on a sand flat, 98.8 m deep, off the east coast of Fuerte Ventura, Canary Islands. The sample was frozen at −20 °C immediately after collection and stored frozen until work-up. The frozen sponge was extracted exhaustively with ethanol:ethyl acetate (EtOH:EtOAc, 1:9 *v/v*) followed by EtOH. After concentration by distillation under reduced pressure, the combined extracts were partitioned between EtOAc and H_2_O. The EtOAc partition was fractionated by MPLC on a Combi-*Flash R*_f_4x using a Redi-Sep Gold C-18 column and a linear gradient of CH_3_CN and H_2_O. Final purification was achieved using preparative HPLC on a Vydac C-18 column, eluted with a linear gradient of CH_3_CN:H_2_O to yield **2** (2.6 mg, 1.1 × 10^−3^% of wet weight) as a colorless oil. The structure was defined through interpretation of high resolution mass spectrometry data coupled with a full 2D NMR data set and confirmed by comparison to the published data (Supporting Figures S26–S34) [14].

### 2.2. Biological Activity

Bebrycin A and nitenin were assayed for their EC_50_ values against the *P. falciparum* chloroquine-resistant strain (Dd2) using a SYBR Green I fluorescence assay. The EC_50_s were determined to be 1.08 ± 0.21 and 0.29 ± 0.02 µM for bebrycin A and nitenin, respectively. To determine the selectivity of these inhibitors for the malaria parasite, cytotoxicity against the HepG2 human hepatocyte carcinoma cell line was evaluated using a formazan based MTS assay. Bebrycin A gave an EC_50_ value against HepG2 of 21.8 ± 1.4 µM for a selectivity index (SI) of 20.1, while nitenin gave an EC_50_ of 18.3 ± 1.1 µM, SI = 62.5.

**Figure 3:**
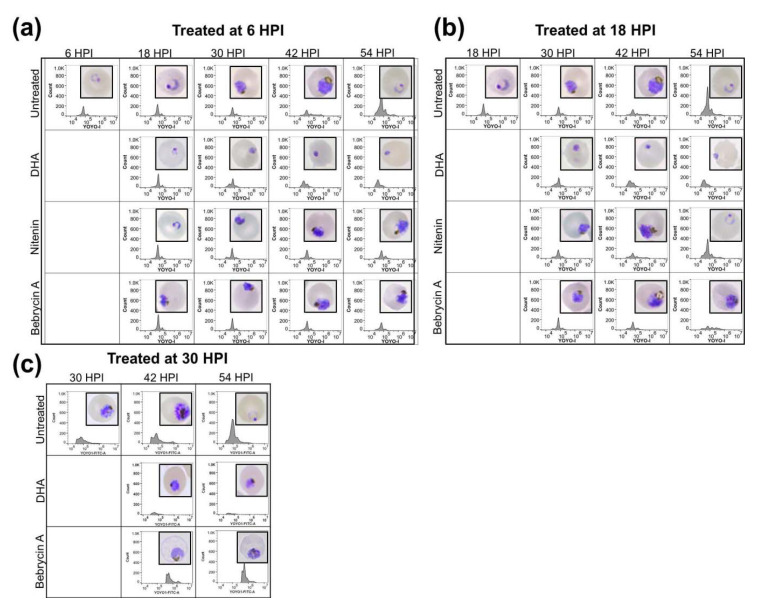
Nitenin and bebrycin A exhibit distinct profiles of inhibition during *P. falciparum* intraerythrocytic maturation. Synchronized Dd2 culture was exposed to the test compounds at 5 × EC_50_ starting at 6, 18, and 30 HPI and monitored into the next life cycle stage up to 54 HPI. Untreated wells (containing DMSO vehicle) or dihydroartemisinin (DHA) were include as controls. Giemsa smears (inset) and flow cytometry with nucleic acid staining fluorophore YOYO-1 were collected every 12 h following compound addition at 6 (**a**), 18 (**b**), and (**c**) 30 HPI. Results represent the combination of three independent replicates.

To better define the mechanism of action, the developmental stage specific effects of the compounds during intraerythrocytic maturation of *P. falciparum* were assessed. Synchronized parasites at the early ring (6 HPI), late ring (18 HPI), and late trophozoite (30 HPI) stages were exposed to a 5 × EC_50_ concentration of compound. The microscopical evaluation of the development stage progression (inset) in addition to the flow cytometric analysis of the DNA content with YOYO-1 was then performed. As is evident in Figure 3, maturation of the nitenin treated culture was inhibited if compound was added early, before the transition of ring to early trophozoite stage (6 HPI). In contrast, exposure of the culture to nitenin at 18 HPI did not impair maturation, and the parasites progressed to the ring stage in the next developmental cycle (54 HPI) similar to the control. Similar results were obtained when parasites were treated at 30 HPI (Figure S35). The early developmental stage specific action of nitenin is significant, as a recent report [17] suggests that only artemisinin and artesunate among antimalarial drugs in clinical use act during the ring stage.

Exposure of the culture to bebrycin A blocked maturation when added at 6, 18, or 30 HPI (Figure 3). Morphologically, parasites treated at 6 HPI did not proceed beyond the late trophozoite-early schizont stages, with no apparent shift in YOYO-1 fluorescence indicative of multinucleation. Treatment at 18 HPI resulted in a schizont like phenotype both morphologically and via the flow cytometric profile. Treatment at 30 HPI also appeared to inhibit during schizogony. However, in contrast to dihydroartemisinin (DHA), which completely inhibits parasite maturation, bebrycin A exposed parasites, although blocked at the schizont stage, appeared to increase in DNA content when treated at 30 HPI. Again, among the current antimalarials, only artemisinin exhibits potent activity at the schizont stage [17]. Therefore, the discovery of nitenin and bebrycin A from marine macro-organisms is significant as these chemotypes act on parasite life cycle stages that are not currently targeted by approved antimalarials other than artemisinin [17].

## 3. Discussion

Previous studies of octocorals belonging to the genus *Bebryce* have led to the isolation of a number of different classes of organic compounds including carotenoids [18], a sterol glycoside [19], and a guaiazulene [20]. Bebrycin A is a diterpene with a rare C-15 membered carbocyclic ring. It can be envisioned as forming from ring opening of the cyclopropyl group of a casbene class terpenoid, or from rearrangement of a cembranoid to yield the 15 membered ring. Recently, a terpene synthase from a marine *Micromonospora* has been identified that produces the diterpene micromonocyclol that has a C-15 membered carbocyclic ring as the direct product of its terpene synthase [21]. Genome mining indicated that this terpene cyclase is common amongst marine *Micromonospora* strains and could potentially be used as a taxonomic marker. Bebrycin A and micromonocyclol are related molecules with different oxidation patterns, and the discovery of this terpene cyclase in marine bacteria opens the possibility that bebrycin A is bacterially produced. Nitenin is a C-21 terpenoid with two beta-substituted furans at distal ends of the molecule. It has been proposed that it is formed through degradation of a larger sesterterpene [13]. Prior reports describing the isolation of nitenin have not reported significant biological activity for nitenin [13,14,22,23,24]. In the current study, we find that both compounds have potent activity against the chloroquine resistant *P. falciparum* strain (Dd2), with good selectivity for the parasite over mammalian cell line HepG2. The specific action of nitenin and bebrycin A on the early or late developmental stages respectively is significant as a recent report [17] suggests that only artemisinin and artesunate among antimalarial drugs in clinical use act on the ring stage and only artemisinin exhibits activity on schizonts [17]. Both bebrycin A and nitenin act on parasite life cycle stages that are not currently targeted by antimalarials other than artemisinin. This may open new avenues for the development of novel classes of antimalarial agents as partner drugs for artemisinin combination therapy.

## 4. Materials and Methods

### 4.1. Chemical Analysis

Optical rotation was measured on a Rudolph Research Analytical AUTOPOL III automatic polarimeter. UV spectra were collected on a NanoDrop Spectrophotometer (Thermo Fisher Scientific, Inc., Waltham, MA, USA). NMR data was collected on a JEOL ECA-600 spectrometer (JEOL USA, Peabody, MA, USA) operating at 600 MHz for ^1^H, and 150.9 for ^13^C. The edited gHSQC spectrum was optimized for 140 Hz and the gHMBC spectrum optimized for 8 Hz. Chemical shifts were referenced to solvent, e.g., CD_3_OD, δ_H_ observed at 3.31 ppm and δ_C_ observed at 49.1 ppm. High-resolution mass spectrometry for **1** was performed on a JEOL AccuTOF-DART 4G (JEOL USA, Peabody, MA, USA) using the ESI source for ionization and detected in positive ion mode. High-resolution mass spectrometry for **2** was performed on a Thermo Fisher Orbitrap (Thermo Fisher Scientific, San Jose, CA, USA) using the ESI source for ionization and detected in positive ion mode. IR data was collected on a Perkin Elmer Spectrum 100 with Universal ATR (Perkin Elmer, Waltham, MA, USA).

### 4.2. Biological Material

Bebrycin A (**1**) was isolated from three separate specimens of the octocoral *Bebryce grandis* (Figures S1 and S2). The primary specimen used in this study was HBOI Sample Number 10-V-00-1-004. A taxonomic reference sample is archived at the FAU Harbor Branch Oceanographic Museum (HBOM) Catalog Number: 012:00825. The specimen was collected off the southeast coast of Curacao, East of Fuikbaai (Latitude: 12 02.265′ N Longitude 68 49.260′ W) in 2000, using the *Johnson-Sea-Link* II submersible at a depth of 128 m. A second specimen that yielded bebrycin A was HBOI Sample Number 18-XI-02-1-005 (HBOM Catalog Number: 012:00823). This specimen was collected in 2002, off the east side of Goulding Cay, New Providence Island, Bahamas (Latitude: 25 00.513′ N Longitude: 77 33.932′ W) using the *Johnson-Sea-Link* I submersible at a depth of 189.6 m. A third specimen that yielded bebrycin A was HBOI Sample Number 11-IV-05-2-001 (HBOM Catalog Number: 012:00824). The specimen was collected in 2005, south of Bimini, Bahamas (Latitude: 25 15.065′ N Longitude 79 10.983′ W) using the *Johnson-Sea-Link* I submersible at a depth of 147.8 m.

All three specimens have external morphology and spicules characteristic of the species *Bebryce grandis* Deichmann, 1936; [Phylum: Cnidaria, Class: Anthozoa, Subclass: Octocorallia, Order: Alcyonacea, Family: Plexauridae]. The species is described in Deichmann (1936) [25], page 125–126, and images are in Bayer and Cairns [26]. The specimens are tan colored, planar with upward curved, stout branches (2 mm diameter), and rounded calyces (1 mm diameter, 1 mm tall), which tend to alternate on the sides. The surface is fine grained, with epifauna including small hollow tubes and Serpulidae worm tubes. The axis is brown and fibrous. The spicules are dominated by cup shaped rosette bodies (0.08 to 0.12 mm tall), and tri-radiate and quad-radiate crosses are about 0.15 to 0.3 mm. It is known from depths of 91 to 281 m, and distribution includes the southeastern U.S., the Gulf of Mexico and the Caribbean.

Nitenin (**2**) was isolated from a sponge (HBOI Sample ID: 5-VI-91-2-007) collected in 1991, using the *Johnson-Sea-Link* I submersible, from a rock outcrop on a sand flat, 98.8 m deep, off the east coast of Fuerte Ventura, Canary Islands [Latitude: 28 09.57’ N, Longitude: 14 06.10’ W] (Figure S24). The morphology is lamellate to fan-shaped, with multiple lamellae arising from a common base. Individual lamellae are up to 15 cm wide, 20 cm high, and 5–10 mm thick. The surface of the sponge is microconulose; abundant oscula (1–2 mm wide, 5–10 mm apart) occur on one side of each lamella and on the other side are only ostia. The sponge was yellow/tan alive. The consistency is dense and compressible. The skeleton is fibroreticulate, dominated by secondary clear fibers (20–30 µm in diameter) and less abundant primary fibers (50–100 µm in diameter) that are cored and covered by sand. Secondary fibers form meshes (100–300 µm in diameter). The specimen has been identified as *Spongia lamella* [Phylum: Porifera, Class Demospongiae Order: Dictyoceratida, Family Spongiidae, Genus/Species *Spongia lamella* (Schulze, 1879) [27] previously known only from the Mediterranean Sea, the Atlantic coast of Portugal and the Straits of Gibraltar [28]. A taxonomic reference sample is archived at the HBOM, catalog number 003:00839.

### 4.3. Extraction and Isolation

Isolation of bebrycin A (**1**): Three different samples were shown to have compound **1**. All samples were frozen at −20 °C immediately after collection and stored frozen until work-up. An example of the isolation process for organism 10-V-00-1-004 follows. The frozen octocoral (133 g) was extracted exhaustively with ethanol (EtOH) followed by ethanol:ethyl acetate ((EtOH: EtOAc)1:9 *v/v*). After concentration by distillation under reduced pressure, the extract (2.9 g) was partitioned between *n*-butanol and H_2_O to yield 0.598 g of butanol partition and 1.7 g of aqueous partition. The *n*-butanol partition was fractionated by medium pressure liquid chromatography (MPLC) on an Isco Combi-*Flash R*_f_4x (Teledyne ISCO, Lincoln, NE, USA) using a Redi-Sep C_18_ column and a linear gradient of CH_3_CN and H_2_O over 20 min. A total of four fractions were subsequently collected. Assay of fraction 3 (34.6 mg) showed activity against *P. falciparum* and 28.2 mg was further fractionated by semi-preparative HPLC on a Vydac C_18_ column (10 × 250 mm, 10 µm particle size) using isocratic elution with CH_3_CN:H_2_O (55:45 *v/v*) to yield **1** (1.9 mg, 1.7 × 10^−3^% of wet weight) eluting at 19 min.

Isolation of nitenin (**2**): A 242 g sample of the frozen sponge, 5-VI-91-2-007, was x extracted exhaustively by macerating with EtOH:EtOAc, (1:9, 2 × 250 mL) followed by EtOH (4 × 200 mL) using a Waring blender and filtered through Celite^®^ (Millipore Sigma, St. Louis, MO, USA). The combined extract was concentrated by distillation under reduced pressure to yield 10.34 g of crude extract. The extract was partitioned between EtOAc and H_2_O (3 × 100 mL portions). The EtOAc partition was concentrated under reduced pressure to yield 1.5 g of a yellow-brown solid. The EtOAc partition was pre-adsorbed onto a small amount of C_18_ stationary phase and then separated by MPLC on an Isco Combi-*Flash R*_f_4x on an Isco RfGold^®^ reverse-phase C_18_ column (26 g) using a linear gradient. The elution gradient contained is as follows: solvent A: 100% water; solvent B: 100% CH_3_CN; t = 0 column volumes (CV), A/B (90:10); t = 2 CV, A/B (90:10); t = 15 CV, A/B (0:100); t = 18 CV, A/B (0:100); flow = 35 mL/min; detected by UV at 280 and 230 nm. Fraction 10 (eluting between 14.5 and 15.5 CV) was further purified by reverse-phase HPLC on a Waters Autopurification system (Milford, MA, USA) using a Vydac Protein and Peptide C_18_ column (25mm × 150 mm, 10 µm particle size), flow rate 15 mL/min; and elution gradient as follows: solvent A: H_2_O:CH_3_CN (95:5 *v/v*), solvent B: 100% CH_3_CN; t = 0 min, A:B (40:60); t = 15 min, 100% B; t = 18 min 100% B; monitored by ESI-MS and UV. The eluent was collected by time, and the tubes from each run were combined accordingly to give nitenin, **2** (2.6 mg, 1.1 × 10^−3^% of frozen weight), eluting at 7.2 min. The structure was defined through interpretation of a full 2D data set (Figures S25–S34) and the data compared with published data [14].

Bebrycin A (**1**); tan oil; [α]_D_^20^ = +5.0 (*c* 0.11 in MeOH); UV (MeOH) λ_max_ (log ε) 220 nm (4.1); ^1^H and ^13^C NMR (Table 1, Figures S4–S22); HRESIMS: C_20_H_32_O_2_ [*m/z* observed 327.2301 [M+Na]^+^, calculated 327.2300, Δ = –0.07 mmu], Figure S23.
Nitenin (**2**); colorless oil; [α]_D_^20^ = -12.8 (*c* 0.054 in MeOH); UV (MeOH) λ_max_ (log ε) 218 nm (3.5); ^1^H and ^13^C NMR (Table S26, Figures S27–S33); HRESIMS: C_21_H_24_O_4_ [*m/z* observed 341.1749 [M + H]^+^, calculated 341.1753, Δ = −0.4 mmu], Figure S34.

### 4.4. Biological Testing

*P. falciparum* culture: The *P. falciparum* chloroquine-resistant Dd2 line was cultured using a modified Trager and Jensen method [29,30]. Briefly, parasite cultures were maintained at 37 °C in 95% air and 5% CO_2_ in RPMI 1640 medium with L-glutamine (Invitrogen-Thermo Fisher) supplemented with 25 mM HEPES, pH 7.4, 26 mM NaHCO_3_, 2% dextrose, 15 mg/L hypoxanthine, 25 mg/L gentamycin, and 0.5% Albumax II (Invitrogen-Thermo Fisher, Waltham, MA, USA).

SYBR Green I Fluorescence Assay: Parasite viability was determined using a SYBR Green I-based fluorescent assay [31,32,33]. Extracts or pure compounds in DMSO were diluted in culture medium and screened at varying concentrations at a maximum DMSO concentration of 0.125%. The diluted fractions/compounds were added to asynchronous Dd2 culture at a 1% parasitemia and 2% hematocrit in black, flat-bottom 96-well plates (Santa Cruz Biotechnology, Dallas, TX, USA). Chloroquine (10 µM) was used as a positive control to determine the baseline fluorescence value. Following a 72 h incubation at 37 °C, the plates were frozen at −80 °C. One hundred microliters of the lysis buffer (20 mM Tris-HCl, 0.08% saponin, 5 mM EDTA, 0.8% Triton X-100, and 0.01% SYBR Green I) was added to each well of the thawed plates. After incubation in the dark at 37 °C for 30 min, the fluorescence emission was measured using a Synergy H4 hybrid multimode plate reader (Biotek, Winooski, VT, USA) set at 485 nm excitation and 530 nm emission. The EC_50_ and SEM were determined using GraphPad Prism 7.0.

Cytotoxicity Assay: Cytotoxicity was determined using the CellTiter Aqueous one solution cell proliferation MTS ([3-(4,5-dimethylthiazol-2-yl)-5-(3-carboxymethoxyphenyl)-2-(4-sulfophenyl)-2H-tetrazolium, inner salt) assay (Promega, Madison, WI, USA). Varying concentrations of fractions/compounds were added to black, clear-bottom 384 well plates, (Santa Cruz Biotechnology) containing HepG2 cells seeded at 2500 cells/well, and incubated in an atmosphere of 5% CO_2_, 95% air at 37 °C for 48 h after seeding. Following incubation, the MTS reagent was added to each well, and the plates were incubated again for 2 h. After, absorbance was recorded for each well using a Biotek Synergy H4 hybrid multimode plate reader at 490 nm. The EC_50_ and SEM were determined using GraphPad Prism 7.0.

Stage Specific Activity Assay (SSA): The SSA was performed as described previously [34]. Briefly, *P. falciparum* Dd2 cultures were tightly synchronized by magnetic isolation of schizonts using MACS (Miltenyi Biotec, Auburn, CA, USA) column [35] followed by 5% sorbitol (*w/v*) treatment [36]. Synchronized cultures were closely monitored by periodic Giemsa staining to identify the time of reinvasion. Six hours post invasion, cultures were plated into a 96 well plate, and compound was added to the 6 HPI treatment wells. At 12 h intervals, intraerythrocytic stage development was monitored via smears for Giemsa staining, and sample collection and fixing for flow cytometry. At each interval, a separate set of wells were likewise treated with compound for 18 and 30 HPI studies. For Giemsa staining of thin smears, a minimum of 1000 RBCs were counted, and the number of ring, trophozoite, and schizont infected RBCs was recorded. The most predominant stage for each smear was imaged using Leica DMi8 microscope and DMX-99 Digital camera.

Flow cytometry was performed on fixed, permeabilized, and YOYO-1 stained samples using CytoFLEX S flow cytometer (Beckman Coulter, Brea, CA, USA) as described previously [37]. Samples were gated to the RBC population and at least 100,000 events per well were recorded.

## Figures and Tables

**Table 1:** ^1^H and ^13^C NMR data for bebrycin A (**1**) (CD_3_OD, 600 MHz).

Position	δ_C_, type	δ_H_ (*J* in Hz)	COSY	HMBC ^1^	NOESY	1D-nOe
1	39.1, qC	-	-	-	-	-
2	157.3, CH	6.83, d (15.8)	3	1, 3, 4, 15, 16	5, 16/17	5, 15ab, 16/17
3	126.9, CH	6.10, d (15.8)	2	1, 4	5, 16/17	5w, 16/17
4	206.7, qC	-	-	-	-	-
5	45.3, CH	2.84, m	6ab, 18	6	-	2, 3, 6abw, 18
6a	35.2, CH_2_	1.59, m	5, 6b, 7ab	-	-	-
6b	-	1.46, m	6, 6a, 7ab	7	-	6a,18w
7a	24.9, CH_2_	1.13, m	6ab, 8ab	-	-	-
7b	-	1.08, m	-	-	-	-
8a	37.7, CH_2_	1.23, m	7a, 8b, 9	7, 19w	-	9w, 19w
8b	-	1.08 m	8a, 9	-	-	-
9	29.3, CH	1.92, m	8ab, 10ab, 19	-	19,8 or 7, 10a	8abw, 10aw, 12bw, 19
10a	48.6, CH_2_	2.59, dd (17.2, 6.2)	9, 10b	8, 9, 11, 19	-	7abw, 12aw, 19
10b	-	2.05, dd (17.2, 6.9)	9, 10a	8, 9, 11, 19	-	8bw, 10a,12aw, 19
11	211.8, qC	-	-	-	-	-
12a	54.5, CH_2_	3.00, d (12.4)	12b	11, 13, 14, 20	14w	10aw, 14w, 20w
12b	-	2.92, d (12.4)	12a	11, 13, 14, 20	14w	14, 20w
13	132.8, qC	-	-	-	-	-
14	126.5, CH	5.22, tq (6.9, 1.4)	15ab, 20	12, 15, 20	12ab, 16/17	2, 12ab, 15ab, 16/ 17
15ab	41.7, CH_2_	2.18, m, 2H	14	1, 2, 13, 14, 16, 17	20, 16/17	14, 16/17, 20
16	27.4, CH_3_	1.12, s	-	1, 2, 15, 17	2, 3, 15ab	2, 3, 14, 15ab
17	26.6, CH_3_	1.08, s	-	1, 2, 15, 16	2, 3, 15ab	-
18	16.7, CH_3_	1.01, d (6.9)	5	4, 5, 6	-	5, 6ab
19	21.3, CH_3_	0.87, d (6.9)	9	8, 9, 10	-	8ab 9,10ab
20	17.3, CH_3_	1.62, s	14	12, 13, 14	15ab	-

^1^*g*HMBC correlations, optimized for 8 Hz, are from proton(s) stated to the carbons listed; w indicates a weak signal.

## Data Availability

Raw NMR jdf files are available upon request from A.E.W. The remaining data is found in the manuscript or the Supplementary Materials.

## Supplementary Materials

The following are available online at https://www.mdpi.com/1660-3397/19/4/179/s1, Figure S1. Description of biological material used in the isolation of bebrycin A; Figure S2. Pictures of *Bebryce grandis* used in the study; Figure S3. Table of NMR data for bebrycin A; Figure S4. ^1^H NMR spectrum of bebrycin A; Figure S5. ^13^C NMR spectrum of bebrycin A; Figures S6 and S7. Expansion of edited g-HSQC spectrum of bebrycin A; Figures S8–S10. Expansion of 2D-DQF-COSY spectrum of bebrycin A; Figures S11–S15. Expansions of 2D-g-HMBC spectrum of bebrycin A; Figures S16 and S17. Expansions of 2D-NOESY spectrum of bebrycin A; Figures S18–S22 1D dpfgse-NOE spectra of bebrycin A; Figure S23 HRESI MS of bebrycin A; Figure S24. Biological material used in the isolation of nitenin; Figure S25. Structure of nitenin with numbering. Figure S26. Comparative Table of ^1^H and ^13^C data published previously for nitenin in CDCl_3_ and data for current isolation in CD_3_OD; Figure S27. ^1^H NMR spectrum of nitenin; Figure S28. Expansion of ^1^H NMR of nitenin with annotation; Figure S29. ^13^C NMR spectrum of nitenin; Figure S30. 2D-DQF-COSY spectrum of nitenin; Figure S31. 2D g-HMBC spectrum of nitenin; Figure S32. 2D-edited g-HSQC spectrum of nitenin; Figure S33. 2D-NOESY spectrum of nitenin; Figure S34. HRESI MS of nitenin; Figure S35. Stage specific interaction of nitenin treated at 30 HPI.
