# Supplementary material for: Antiplasmodial Compounds from Deep-Water Marine Invertebrates
Source: Mar Drugs. 2021 Mar 25;19(4):179. doi: 10.3390/md19040179 (PMC8064351; doi:10.3390/md19040179)

Supplementary material for:

ANTIPLASMODIAL COMPOUNDS FROM DEEP-WATER MARINE INVERTEBRATES

Jennifer E. Collins[2], Bracken Roberts[2], Jill C. Roberts[1], Priscilla L. Winder[1], John K. Reed[1], M. Cristina Diaz[1], Shirley A. Pomponi[1], Debopam Chakrabarti[2]*, and Amy E. Wright[1]*

[1] Marine Biomedical and Biotechnology Program, Harbor Branch Oceanographic Institute, Florida Atlantic University. 5600 US 1 North, Ft. Pierce, Florida 34946

[2] Burnett School of Biomedical Sciences, University of Central Florida, Orlando Florida, United States

[*] Correspondence: awrigh33@fau.edu; Tel: +1-772-242-2459; dchak@ucf.edu; Tel: +1-407-882-2256

S1. Description of biological material used in the isolation of bebrycin A (**1**).
S2. Pictures of *Bebryce grandis* used in the study
S3. Table of NMR data for bebrycin A in methanol-d_4 (600 MHZ).
S4. ^{1}H NMR spectrum of bebrycin A (600 MHz methanol-d_4).
S5. ^{13}C NMR spectrum of bebrycin A (150 MHz methanol-d_4).
S6. Expansion of edited g-HSQC spectrum of bebrycin A (150 MHz methanol-d_4).
S7. Expansion of edited g-HSQC spectrum of bebrycin A (150 MHz methanol-d_4).
S8. Expansion of 2D-DQF-COSY spectrum of bebrycin A (600 MHz methanol-d_4).
S9. Expansion of 2D-DQF-COSY spectrum of bebrycin A (600 MHz methanol-d_4).
S10. Expansion of 2D-DQF-COSY spectrum of bebrycin A (600 MHz methanol-d_4).
S11. Expansion of 2D-g-HMBC spectrum of bebrycin A (150 MHz methanol-d_4).
S12. Expansion of 2D-g-HMBC spectrum of bebrycin A (150 MHz methanol-d_4).
S13. Expansion of 2D-g-HMBC spectrum of bebrycin A (150 MHz methanol-d_4).
S14. Expansion of 2D-g-HMBC spectrum of bebrycin A (150 MHz methanol-d_4).
S15. Expansion of 2D-g-HMBC spectrum of bebrycin A (150 MHz methanol-d_4).
S16. Expansion of 2D-NOESY spectrum of bebrycin A (600 MHz methanol-d_4).
S17. Expansion of 2D-NOESY spectrum of bebrycin A (600 MHz methanol-d_4).
S18. 1D dpfgse-NOE spectrum of bebrycin A (600 MHz, methanol-d_4) irradiated at H-5.
S19. 1D dpfgse NOE spectrum of bebrycin A (600 MHz, methanol-d_4) irradiated at H-18.
S20. 1D dpfgse NOE spectrum of bebrycin A (600 MHz, methanol-d_4) irradiated at H-14.
S21. 1D dpfgse NOE spectrum of bebrycin A (600 MHz, methanol-d_4) irradiated at H-2.
S22. 1D dpfgse-NOE spectrum of bebrycin A (600 MHz, methanol-d_4) irradiated at H-3.
S23. HRESI MS of bebrycin A.
S24. Description of biological material used in the isolation of nitenin.
S25. Structure of nitenin (**2**) with numbering.
S26. Table of ^{1}H and ^{13}C data showing published data for nitenin in $CDCl_3$ and current isolation in CD_3OD, 600 MHz.
S27. ^{1}H NMR spectrum of nitenin (**2**) (600 MHz, methanol-d_4).
S28. Expansion of ^{1}H NMR of nitenin (**2**) (600 MHz, methanol-d_4).
S29. ^{13}C spectrum of nitenin (**2**) (150 MHz, methanol-d_4).
S30. 2D-DQF-COSY spectrum of nitenin (**2**) (600 MHz, methanol-d_4).
S31. 2D g-HMBC spectrum of nitenin (**2**) (150 MHz, methanol-d_4).
S32. 2D-edited gHSQC spectrum of nitenin (**2**) (150 MHz, methanol-d_4).
S33. 2D- NOESY spectrum of nitenin (**2**) (600 MHz, methanol-d_4).
S34. HR ESI Mass Spectrum for nitenin (**2**).
S35. Stage specific interaction of nitenin (**2**) during *P. falciparum* intraerythrocytic maturation treated at 30 hours post invasion (HPI)

S1. Biological material used in the isolation of bebrycin A (**1**).

Bebrycin A was isolated from three separate specimens of the octocoral *Bebryce grandis*. The primary specimen used in this study was HBOI Sample Number: 10-V-00-1-004/HBOI Museum Catalog Number: 012:00825. The specimen was collected off the southeast coast of Curacao, east of Fuikbaai (Latitude: 12 02.265′N Longitude: 68 49.260′W) using the Johnson Sea Link II human occupied submersible at a depth of 128 m. A second specimen that yielded bebrycin A was HBOI Sample Number: 18-XI-02-1-005/HBOI Museum Catalog Number: 012:00823. This specimen was collected off the east side of Goulding Cay, New Providence Island, Bahamas (Latitude: 25 00.513′ N Longitude: 77 33.932′ W) using the Johnson Sea Link I human occupied submersible at a depth of 189.6 m. A third specimen that yielded bebrycin A was HBOI Sample Number: 11-IV-05-2-001/HBOI Museum Catalog Number: 012:00824. The specimen was collected south of Bimini, Bahamas (Latitude: 25 15.065′ N Longitude: 79 10.983′ W) using the Johnson Sea Link I human occupied submersible at a depth of 147.8 m.

All three specimens have external morphology and spicules characteristic of the species *Bebryce grandis* Deichmann, 1936; Cnidaria (Phylum), Anthozoa (Class), Octocorallia (Subclass), Alcyonacea (Order), Holaxonia (Suborder), Plexauridae (Family). The species is described in Deichmann (1936), page 125-126, and images are in Bayer and Cairns (2004). The specimens are tan colored, planar with upward curved, stout branches (2 mm diameter), and rounded calyces (1 mm diameter, 1 mm tall), which tend to alternate on the sides. The surface is fine grained, with epifauna including small hollow tubes and Serpulidae worm tubes. The axis is brown and fibrous. The spicules are dominated by cup shaped rosette bodies (0.08 to 0.12 mm tall), and tri-radiate and quad-radiate crosses are about 0.15 to 0.3 mm. It is known from depths of 91 to 281 m, and distribution includes the southeastern U.S., the Gulf of Mexico, and the Caribbean.

References for Taxonomic ID of *Bebryce* Specimens:

Bayer, F.M. & Cairns, S.D. (Eds.) (2004) *The unpublished plates for A.E. Verrill's unfinished report on the Alcyonaria of the "Blake" expeditions: with revised explanations of the figures transcribed from A.E. Verrill's original typescript*. Department of Zoology, National Museum of Natural History, Washington, D.C, viii pp. + 156 pls.

Deichmann, E (1936) The Alcyonaria of the western part of the Atlantic Ocean. *Memoirs of the Museum of Comparative Zoology* 53:1-317 + 37pls

S2. Pictures of 10-V-00-1-004 *Bebryce grandis.*

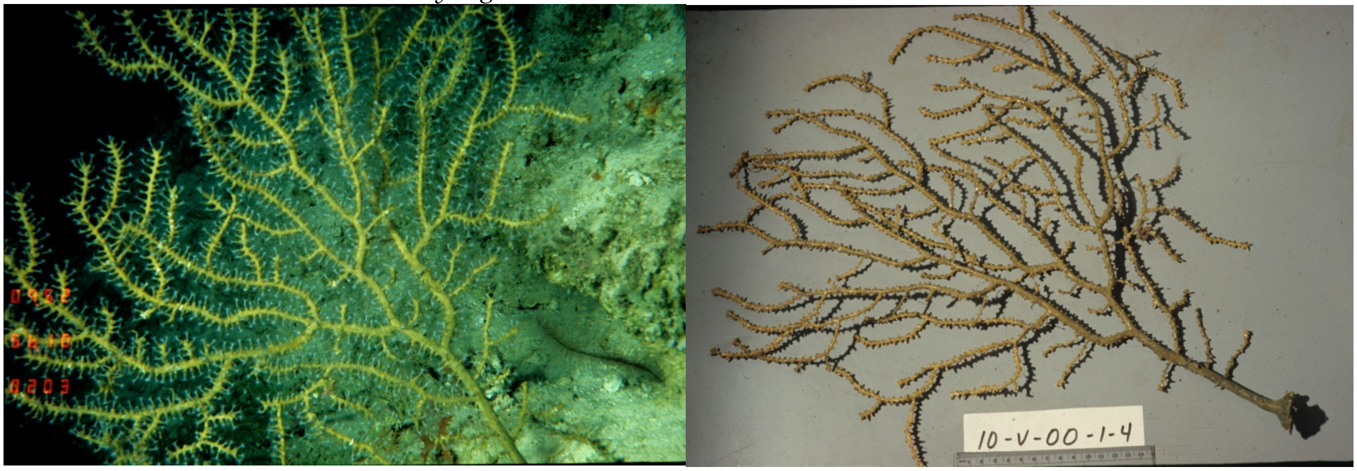

Pictures of 18-XI-02-1-005 *Bebryce grandis.*

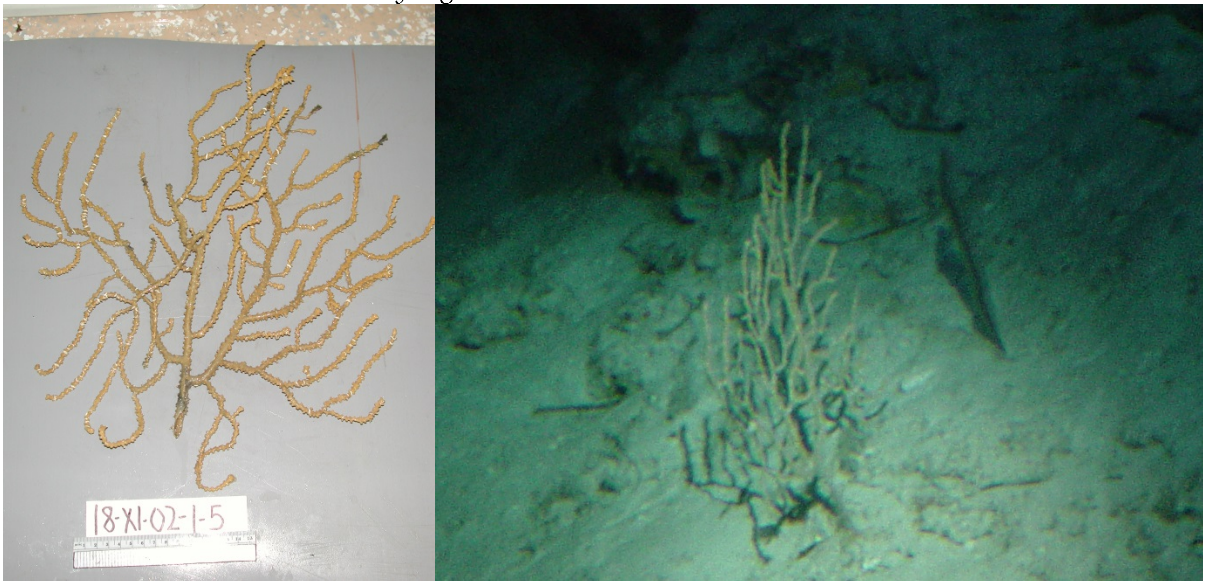

Pictures of 11-IV-05-2-001 *Bebryce grandis.*

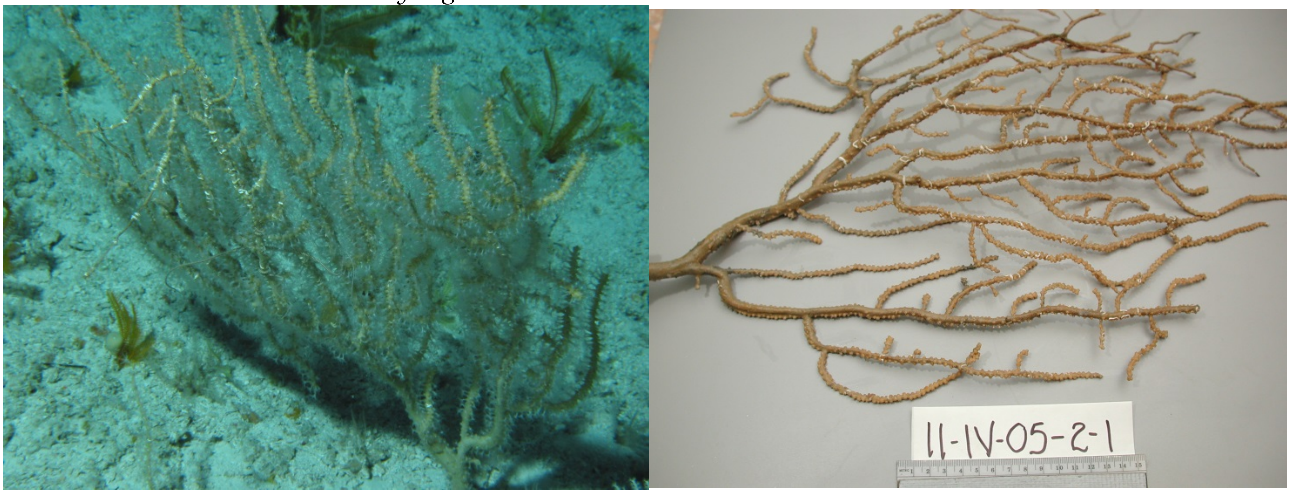

S3. Table of NMR data for bebrycin A in methanol-d_4 (600 MHz).

Position	δ_C, type	δ_H (J in Hz)	COSY	HMBC[1]	NOESY	1D-nOe
1	39.1, qC	-	-	-	-	-
2	157.3, CH	6.83, d (15.8)	3	1, 3, 4, 15, 16	5, 16/17	5, 15ab, 16/17
3	126.9, CH	6.10, d (15.8)	2	1, 4	5, 16/17	5w, 16/17
4	206.7, qC	-	-	-	-	-
5	45.3, CH	2.84, m	6ab, 18	6	-	2, 3, 6abw, 18
6a	35.2, CH_2	1.59, m	5, 6b, 7ab	-	-	-
6b	-	1.46, m	6, 6a, 7ab	7	-	6a,18w
7a	24.9, CH_2	1.13, m	6ab, 8ab	-	-	-
7b	-	1.08, m	-	-	-	-
8a	37.7, CH_2	1.23, m	7a, 8b, 9	7, 19w	-	9w, 19w
8b	-	1.08 m	8a, 9	-	-	-
9	29.3, CH	1.92, m	8ab, 10ab, 19	-	19,8 or 7, 10a	8abw,10aw,12bw,19
10a	48.6, CH_2	2.59, dd (17.2, 6.2)	9, 10b	8, 9, 11, 19	-	7abw,12aw,19
10b	-	2.05, dd (17.2, 6.9)	9, 10a	8, 9, 11, 19	-	8bw, 10a,12aw,19
11	211.8, qC	-	-	-	-	-
12a	54.5, CH_2	3.00, d (12.4)	12b	11, 13, 14, 20	14w	10aw, 14w, 20w
12b	-	2.92, d (12.4)	12a	11, 13, 14, 20	14w	14, 20w
13	132.8, qC	-	-	-	-	-
14	126.5, CH	5.22, tq (6.9, 1.4)	15ab, 20	12, 15, 20	12ab, 16/17	2, 12ab, 15ab, 16/ 17
15ab	41.7, CH_2	2.18, m, 2H	14	1, 2, 13, 14, 16, 17	20, 16/17	14, 16/17, 20
16	27.4, CH_3	1.12, s	-	1, 2, 15, 17	2, 3, 15ab	2, 3, 14, 15ab
17	26.6, CH_3	1.08, s	-	1, 2, 15, 16	2, 3, 15ab	-
18	16.7, CH_3	1.01, d (6.9)	5	4, 5, 6	-	5, 6ab
19	21.3, CH_3	0.87, d (6.9)	9	8, 9, 10	-	8ab 9,10ab
20	17.3, CH_3	1.62, s	14	12, 13, 14	15ab	-

[1]*g*HMBC correlations, optimized for 8 Hz, are from proton(s) stated to the carbons listed; w indicates a weak signal.

S4. ^{1}H NMR spectrum of bebrycin A (600 MHz, methanol-*d*4).

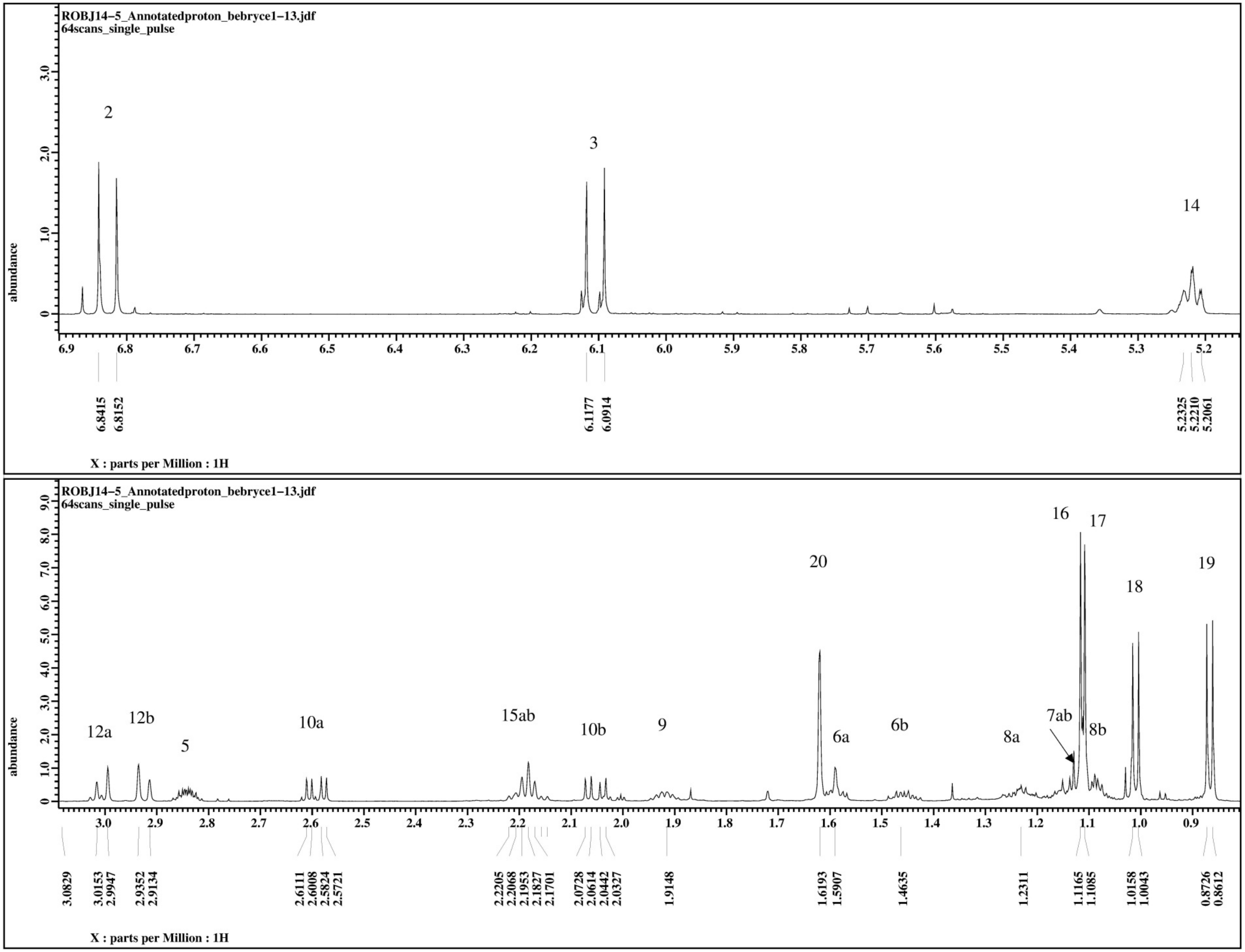

S5. ^{13}C NMR spectrum of bebrycin A (150 MHz, methanol-d_4).

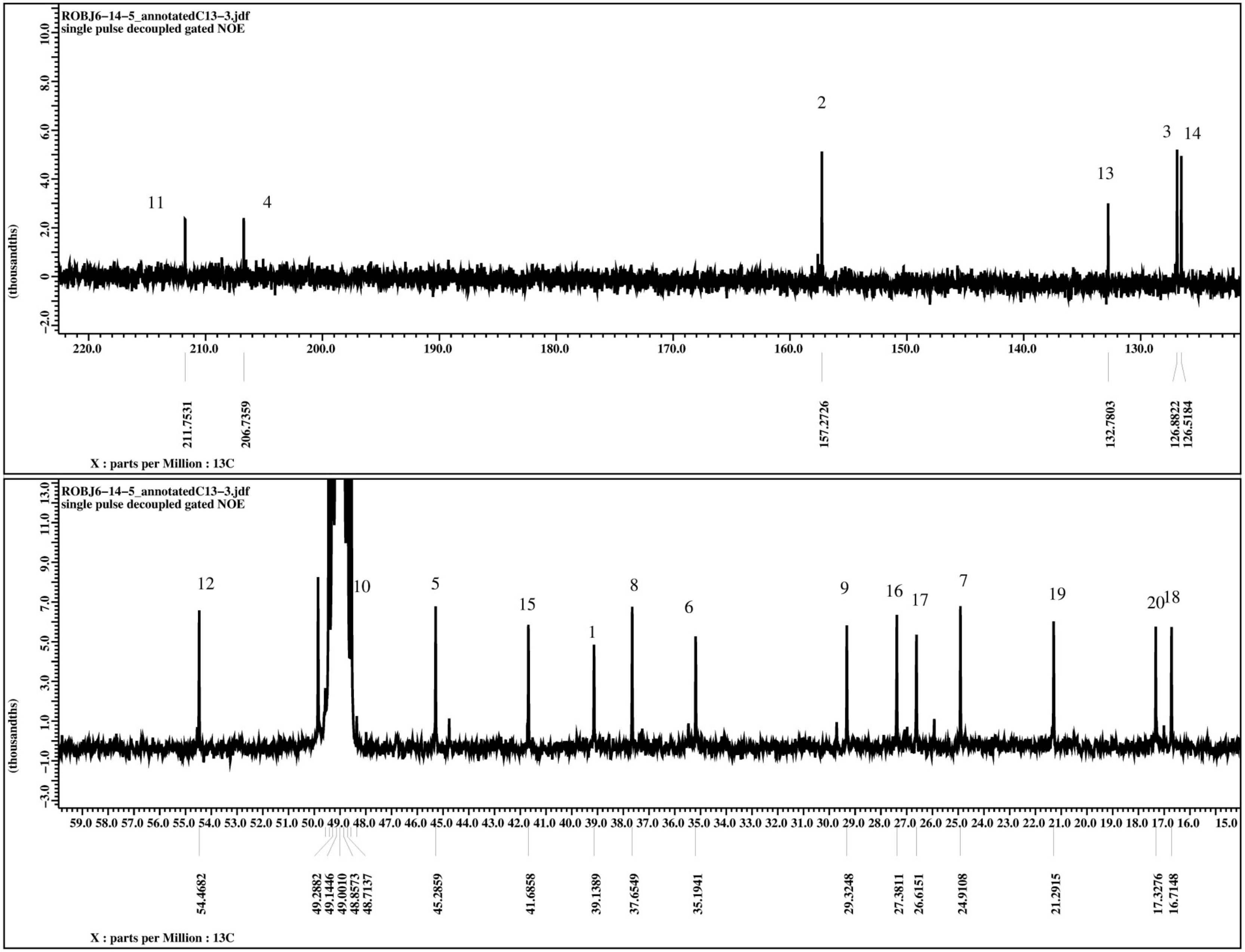

S6. Expansion of edited g-HSQC spectrum of bebrycin A (150 MHz, methanol-d_4).

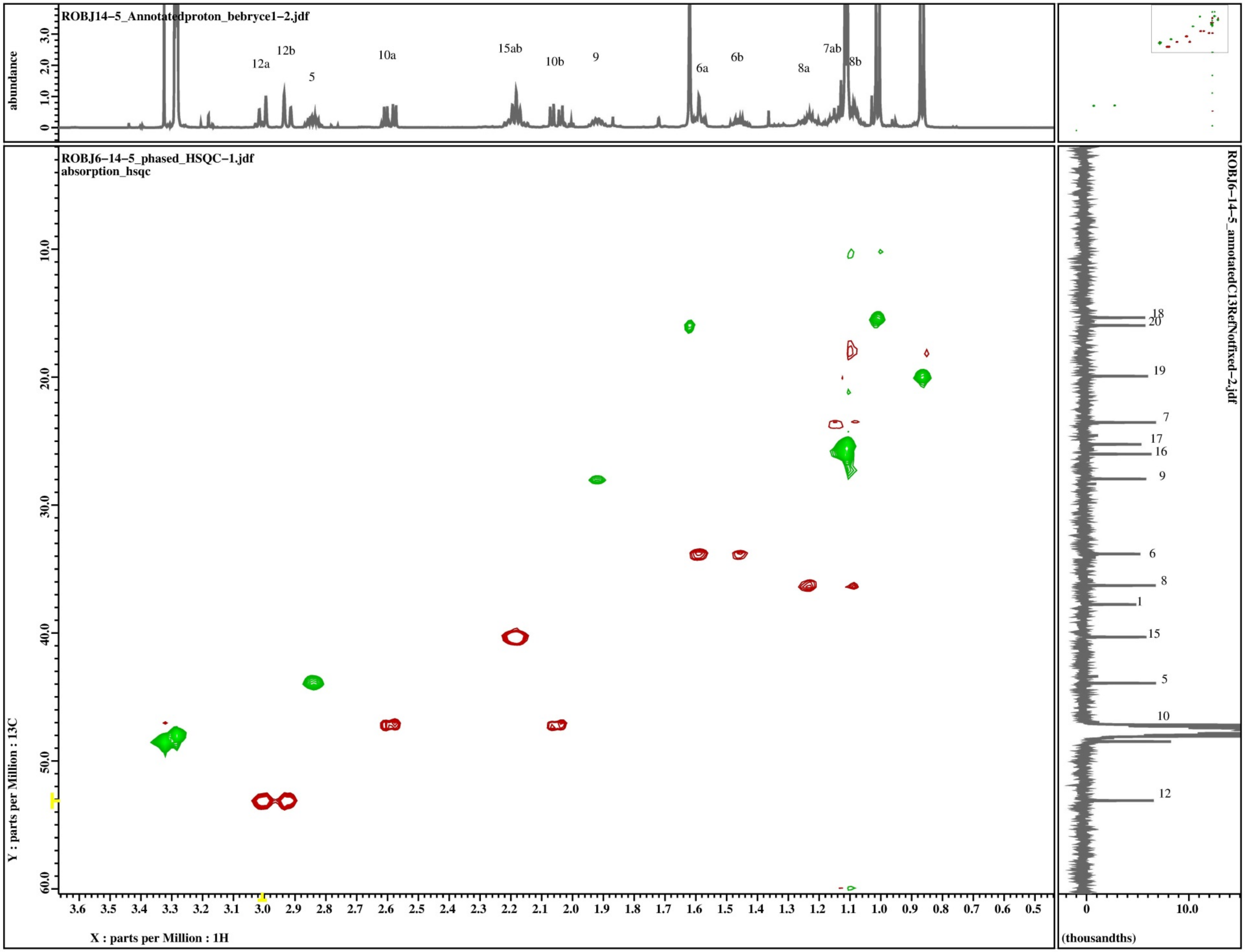

S7. Expansion of edited g-HSQC spectrum of bebrycin A (150 MHz, methanol-d_4).

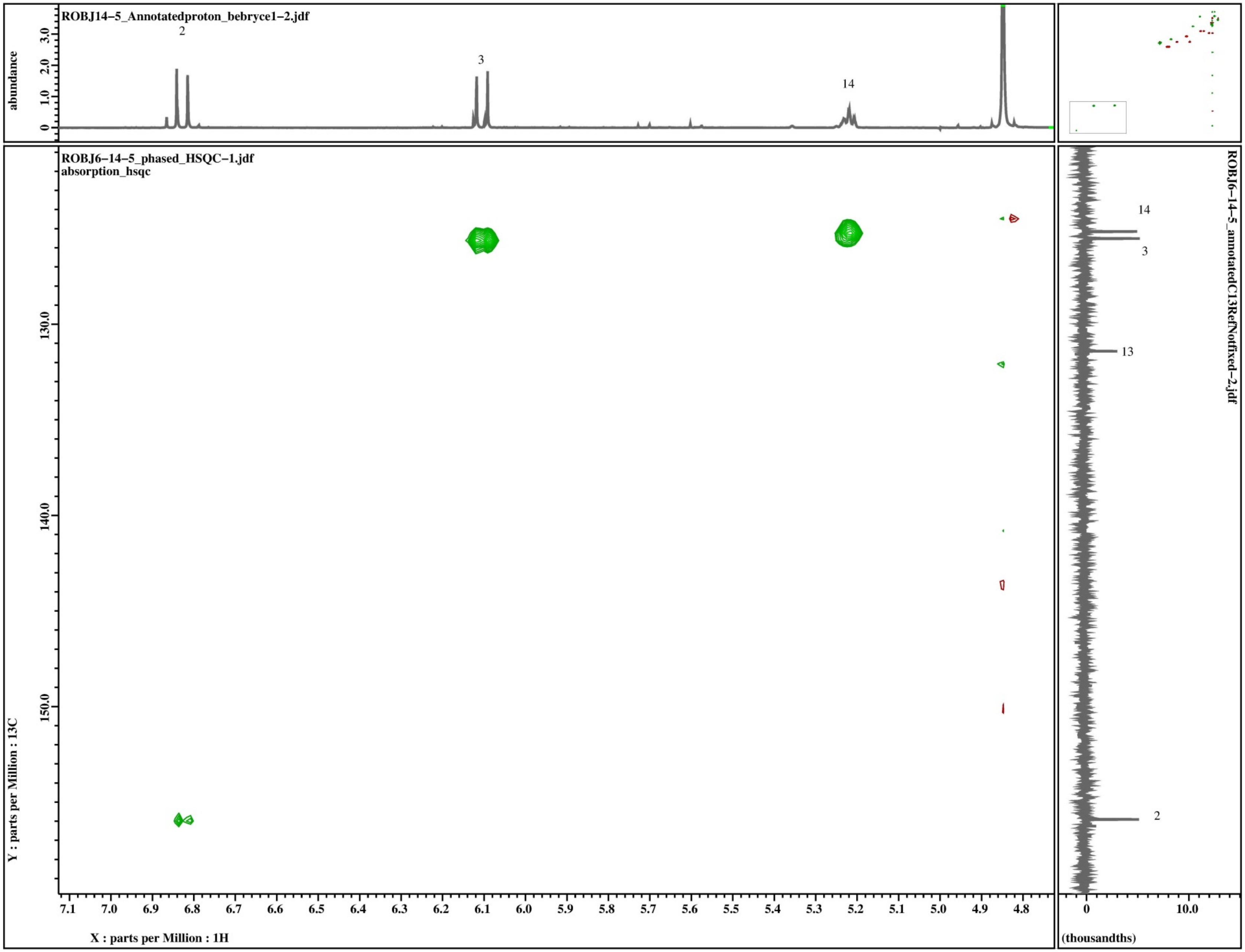

S8. Expansion of 2D-DQF-COSY spectrum of bebrycin A (600 MHz, methanol-d_4).

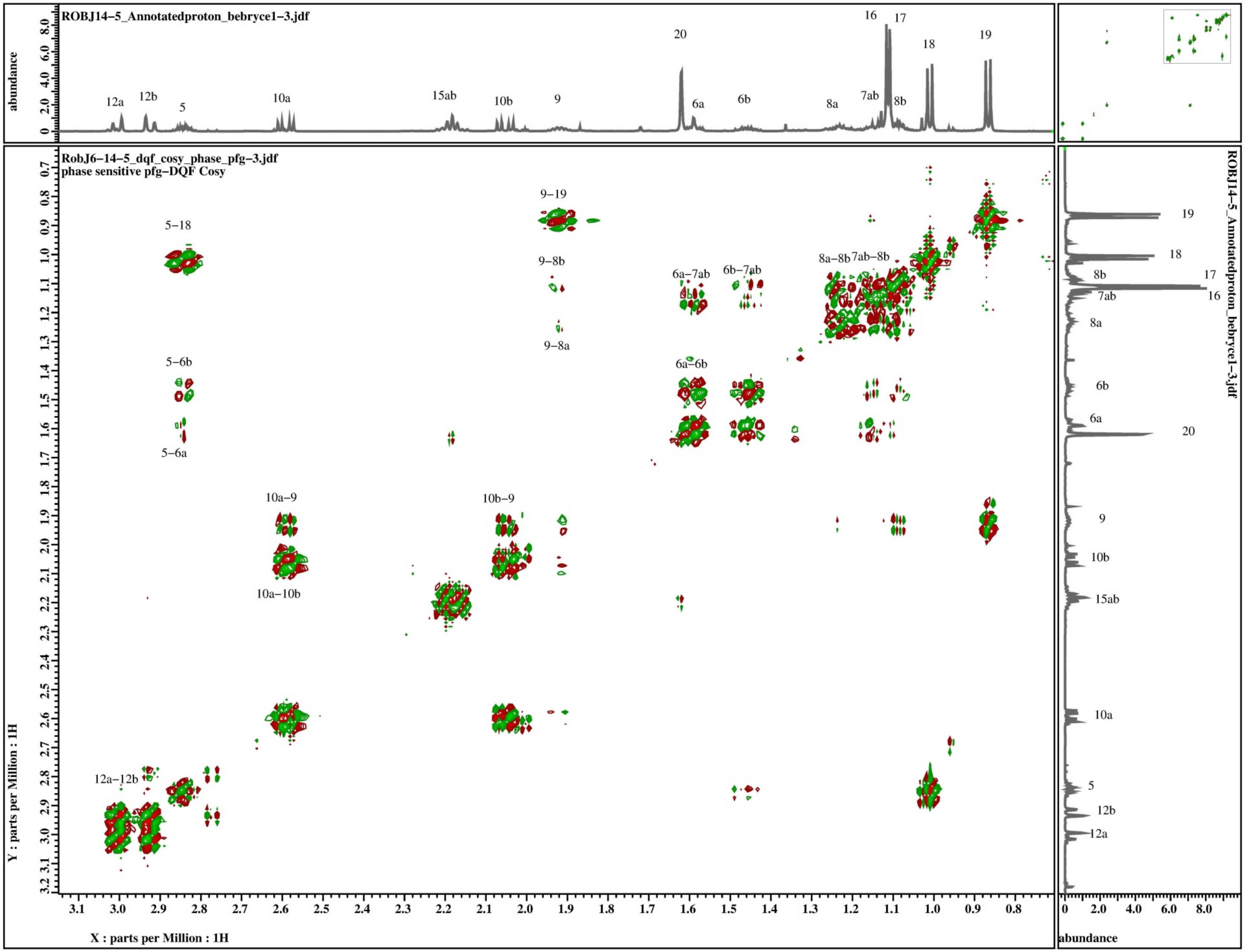

S9. Expansion of 2D-DQF-COSY spectrum of bebrycin A (600 MHz, methanol-d_4).

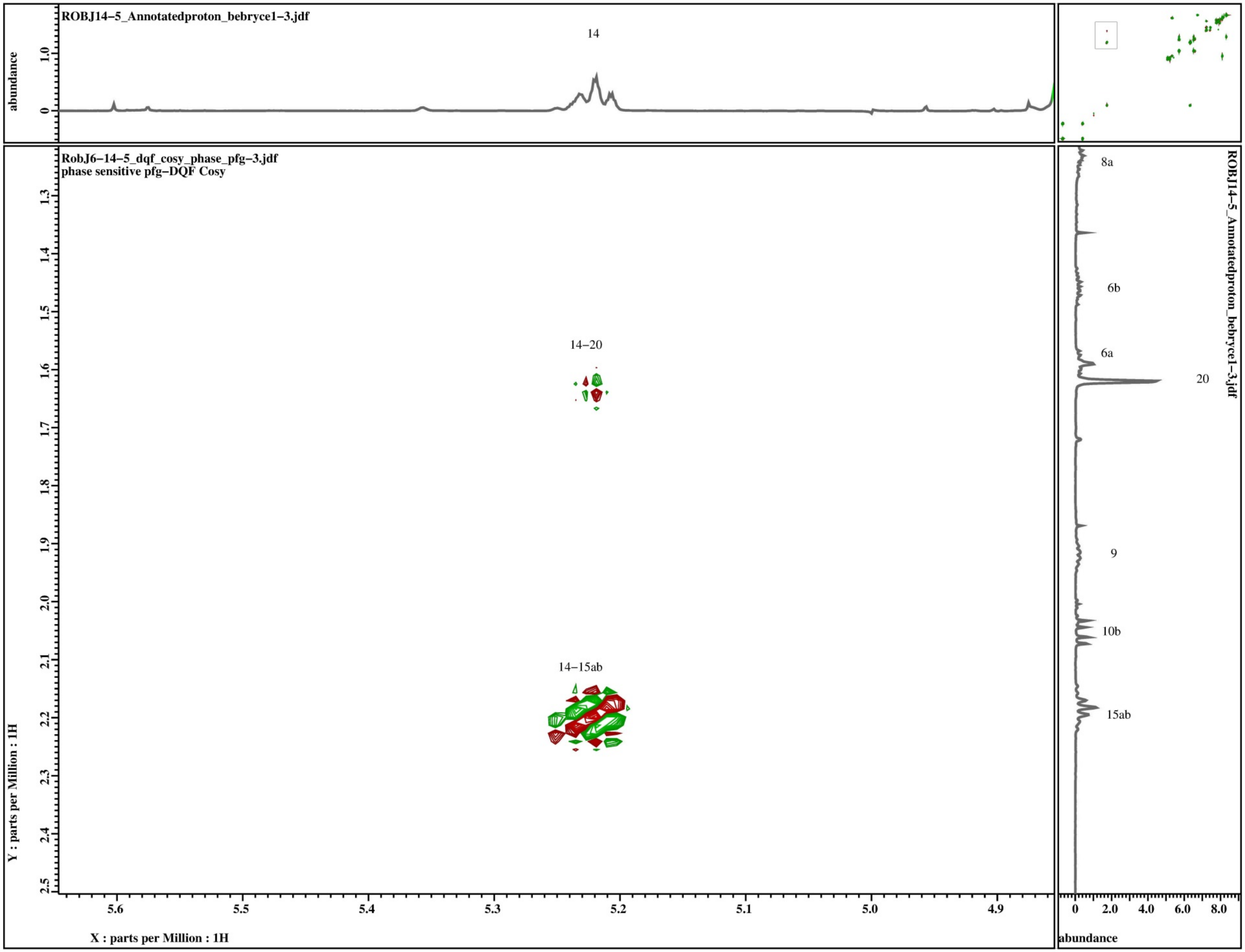

S10. Expansion of 2D-DQF-COSY spectrum of bebrycin A (600 MHz, methanol-d_4).

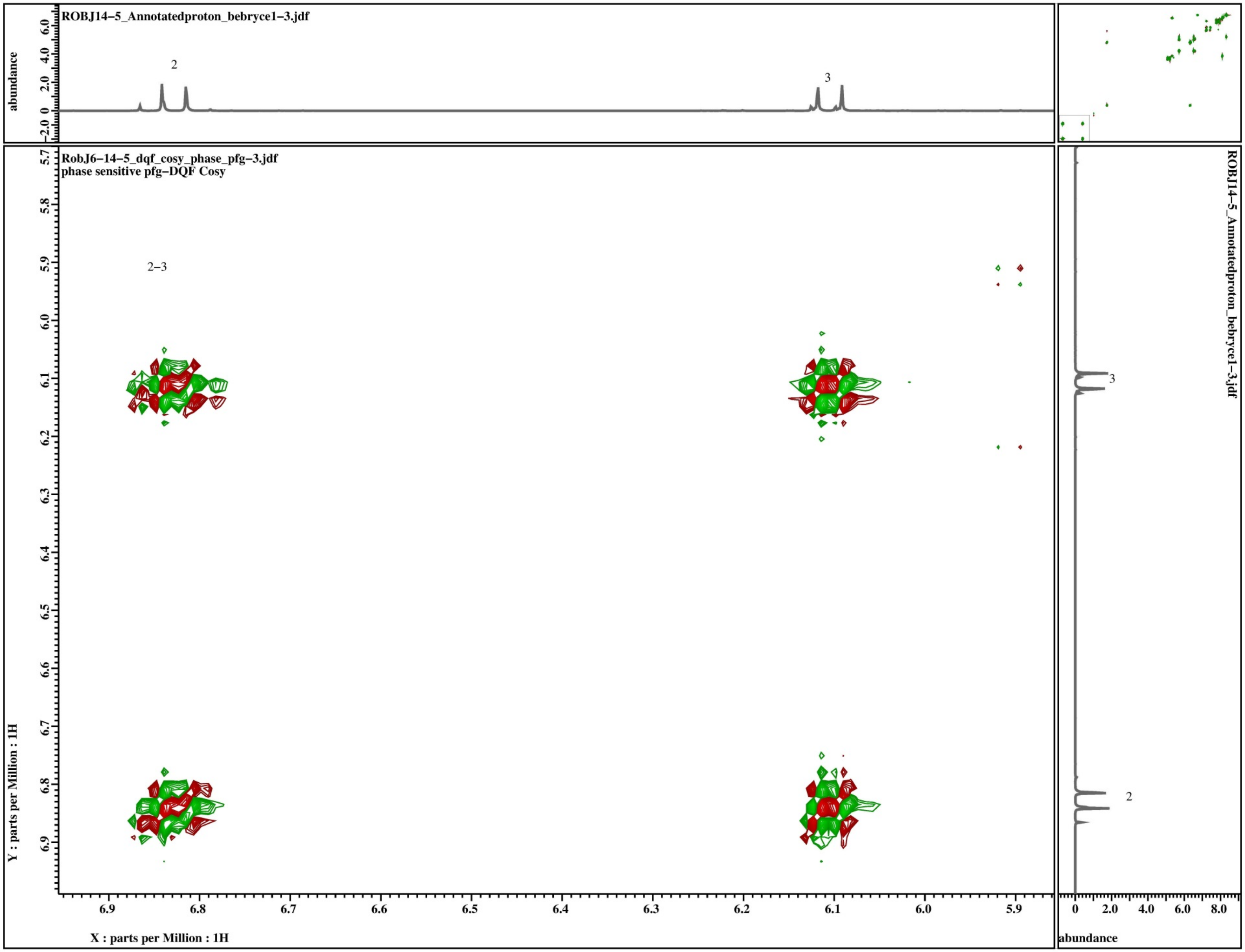

S11. Expansion of 2D-g-HMBC spectrum of bebrycin A (600 MHz, methanol-d_4).

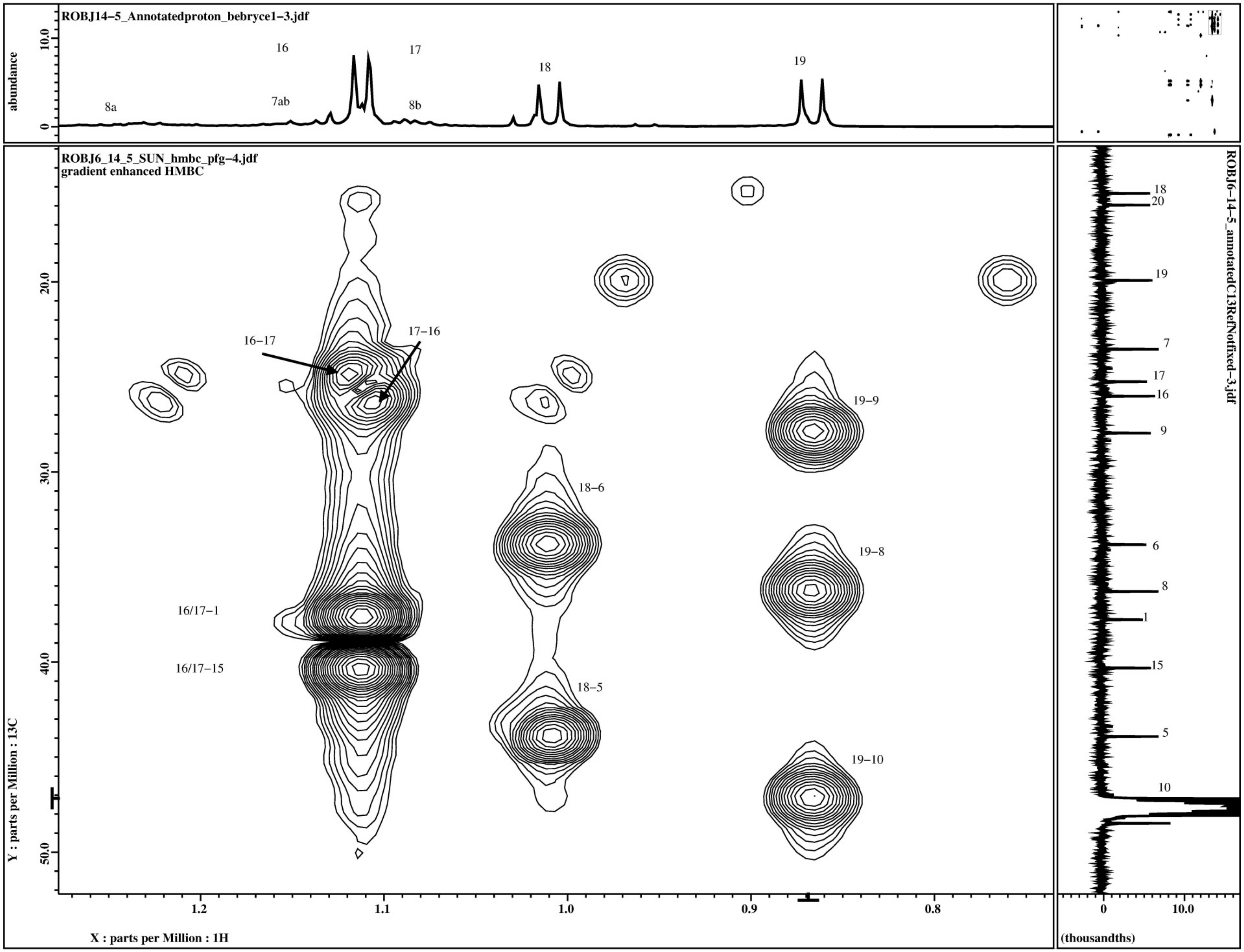

S12. Expansion of 2D-g-HMBC spectrum of bebrycin A (600 MHz, methanol-d_4).

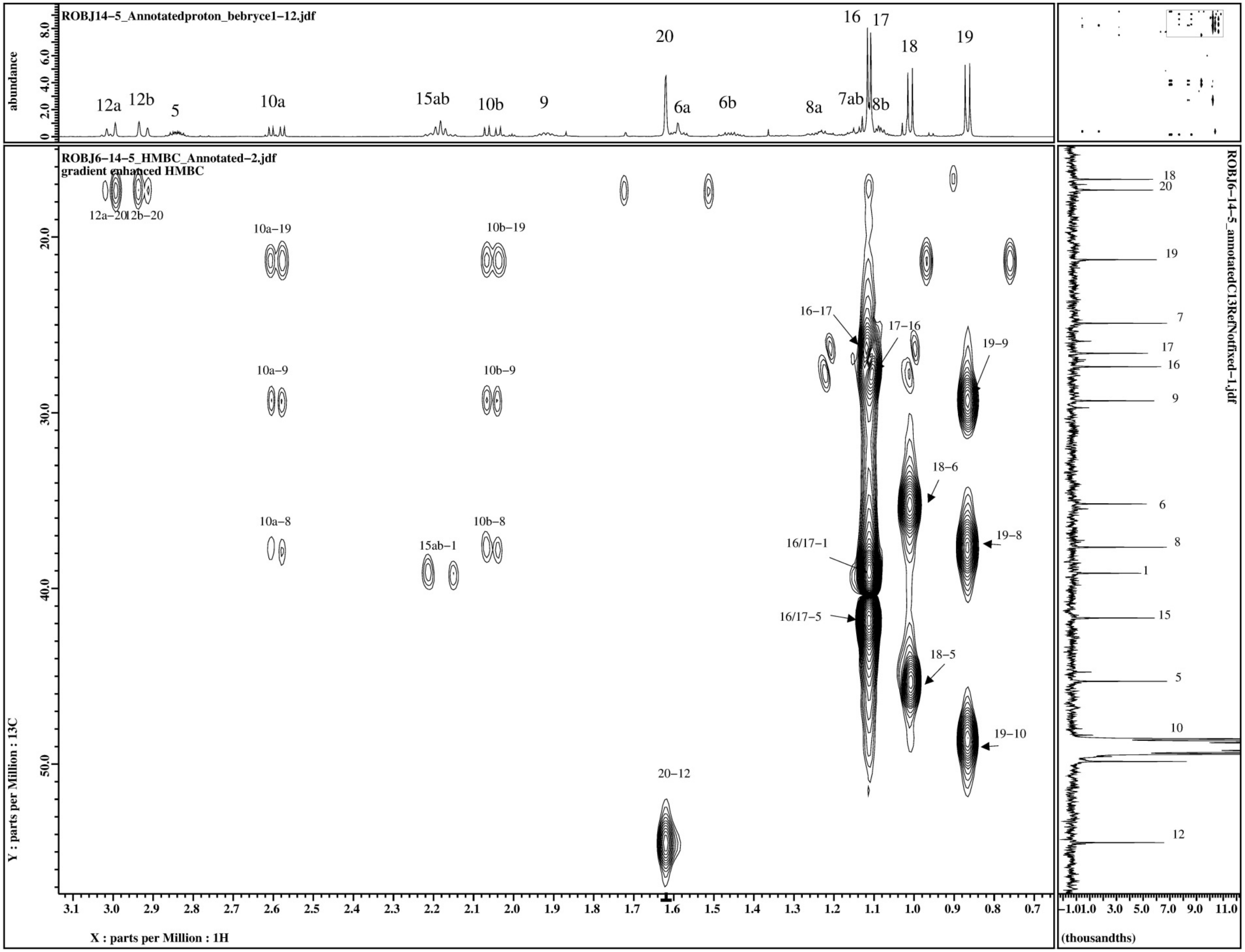

S13. Expansion of 2D-g-HMBC spectrum of bebrycin A (150 MHz, methanol-d_4).

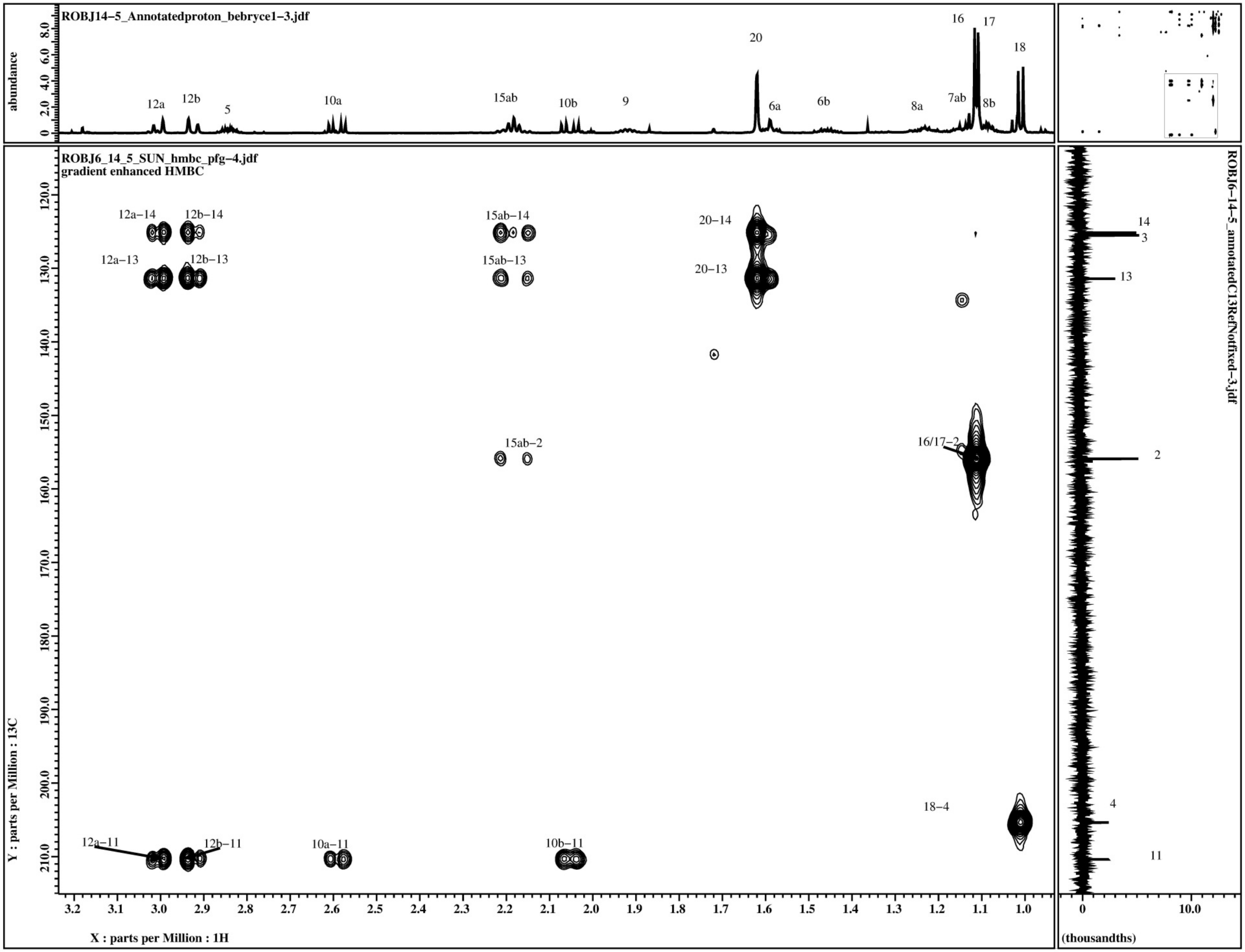

S14. Expansion of 2D-g-HMBC spectrum of bebrycin A (150 MHz, methanol-d_4).

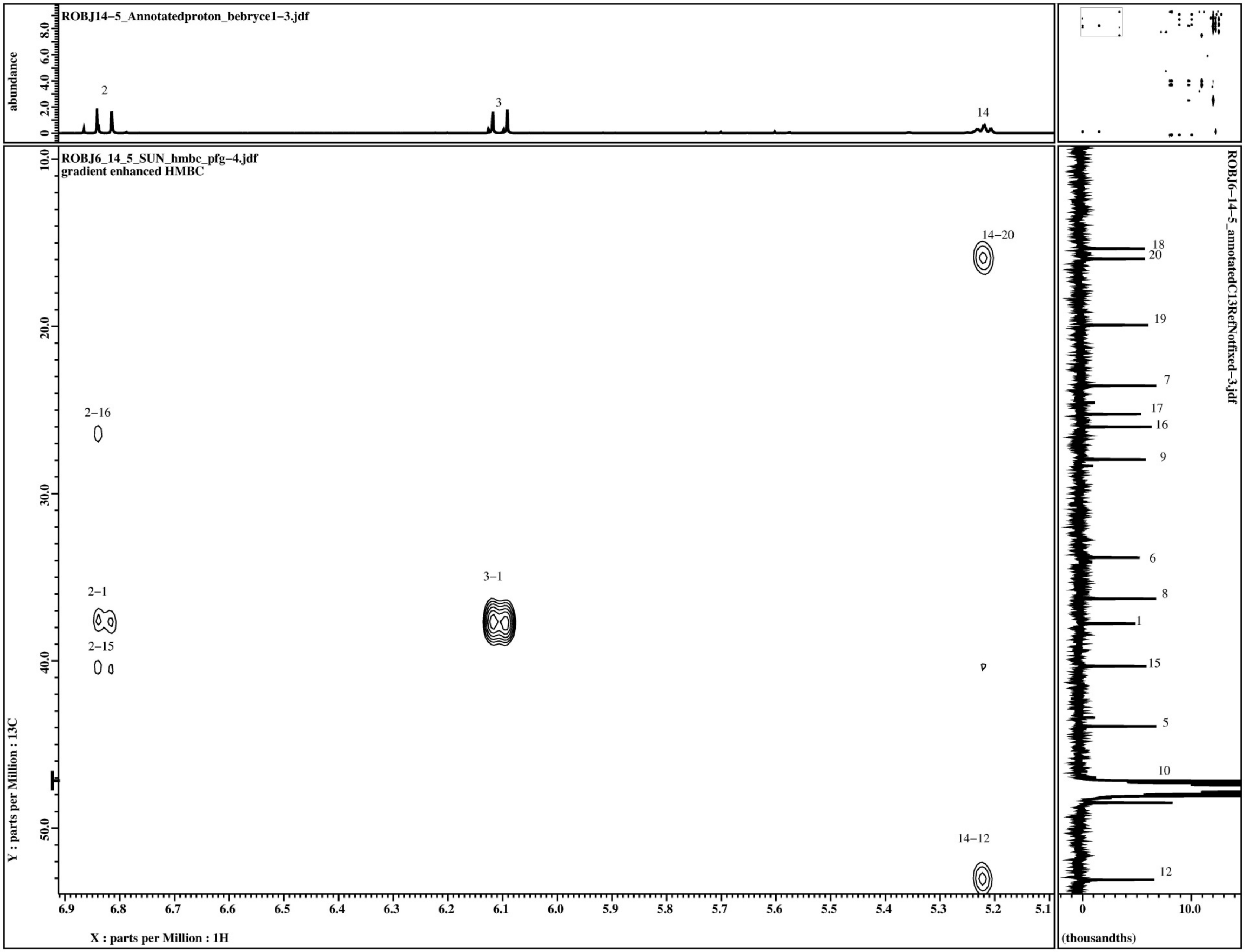

S15. Expansion of 2D-g-HMBC spectrum of bebrycin A (150 MHz, methanol-d_4).

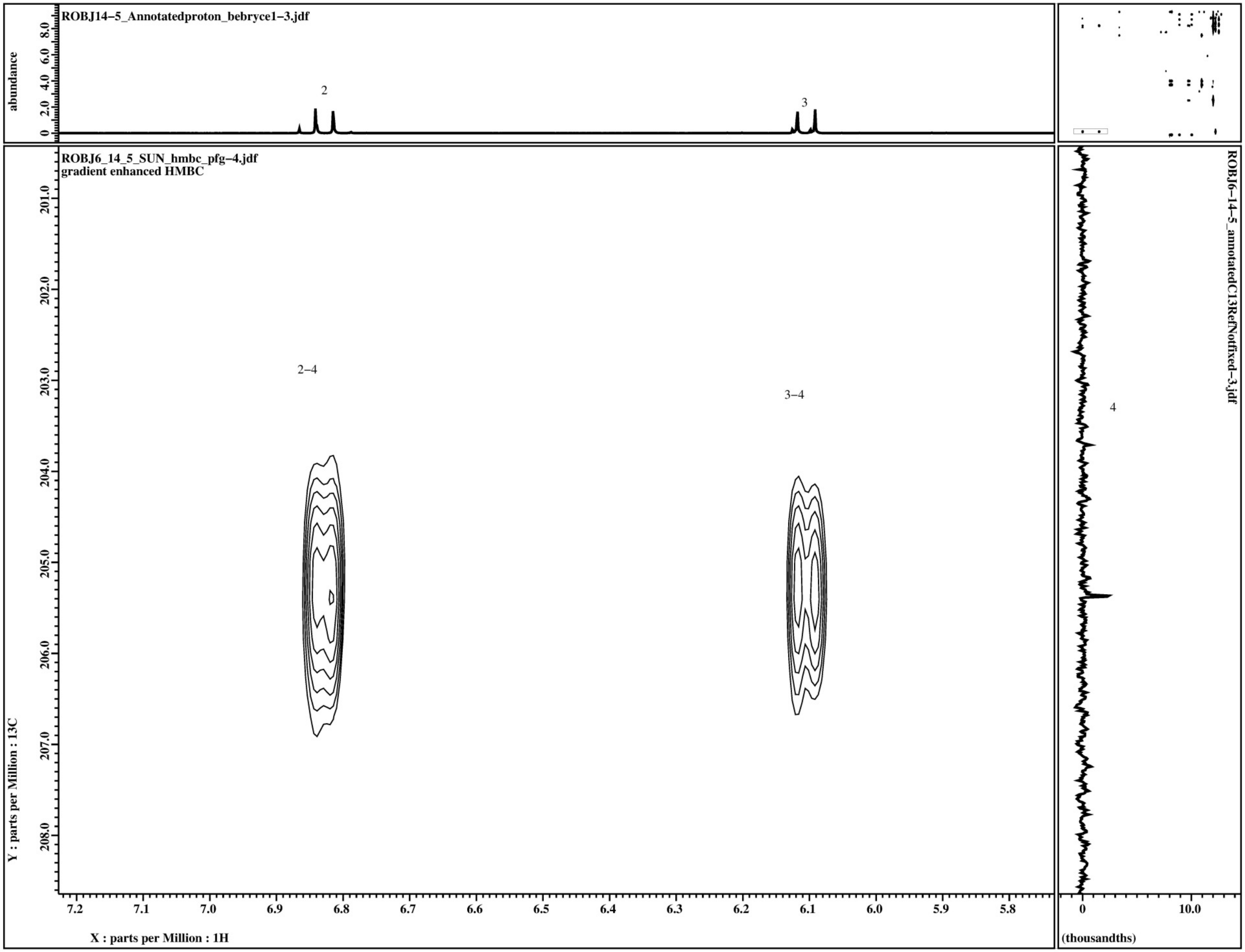

S16. Expansion of 2D-NOESY spectrum of bebrycin A (600 MHz, methanol-d_4).

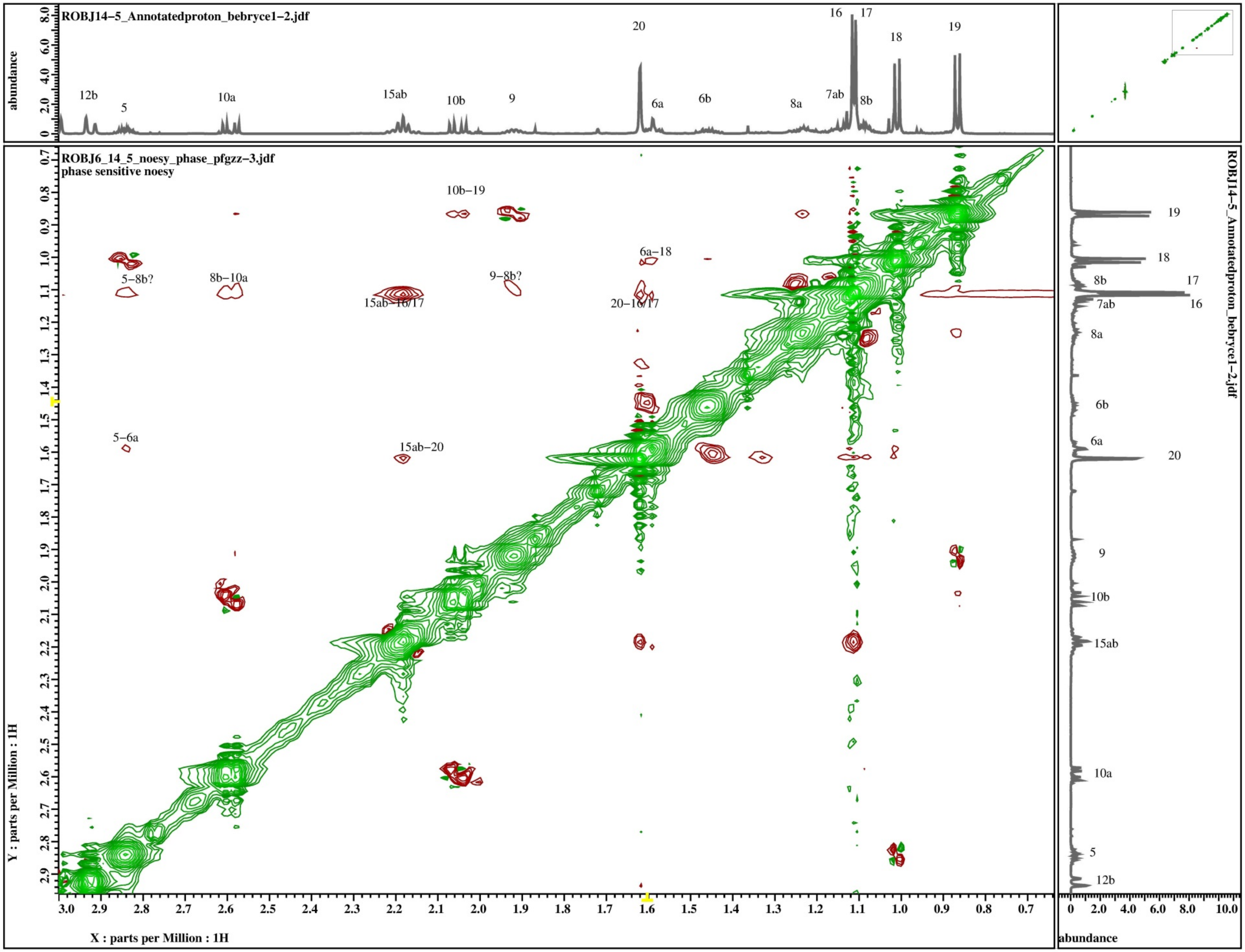

S17. Expansion of 2D-NOESY spectrum of bebrycin A (600 MHz, methanol-d_4).

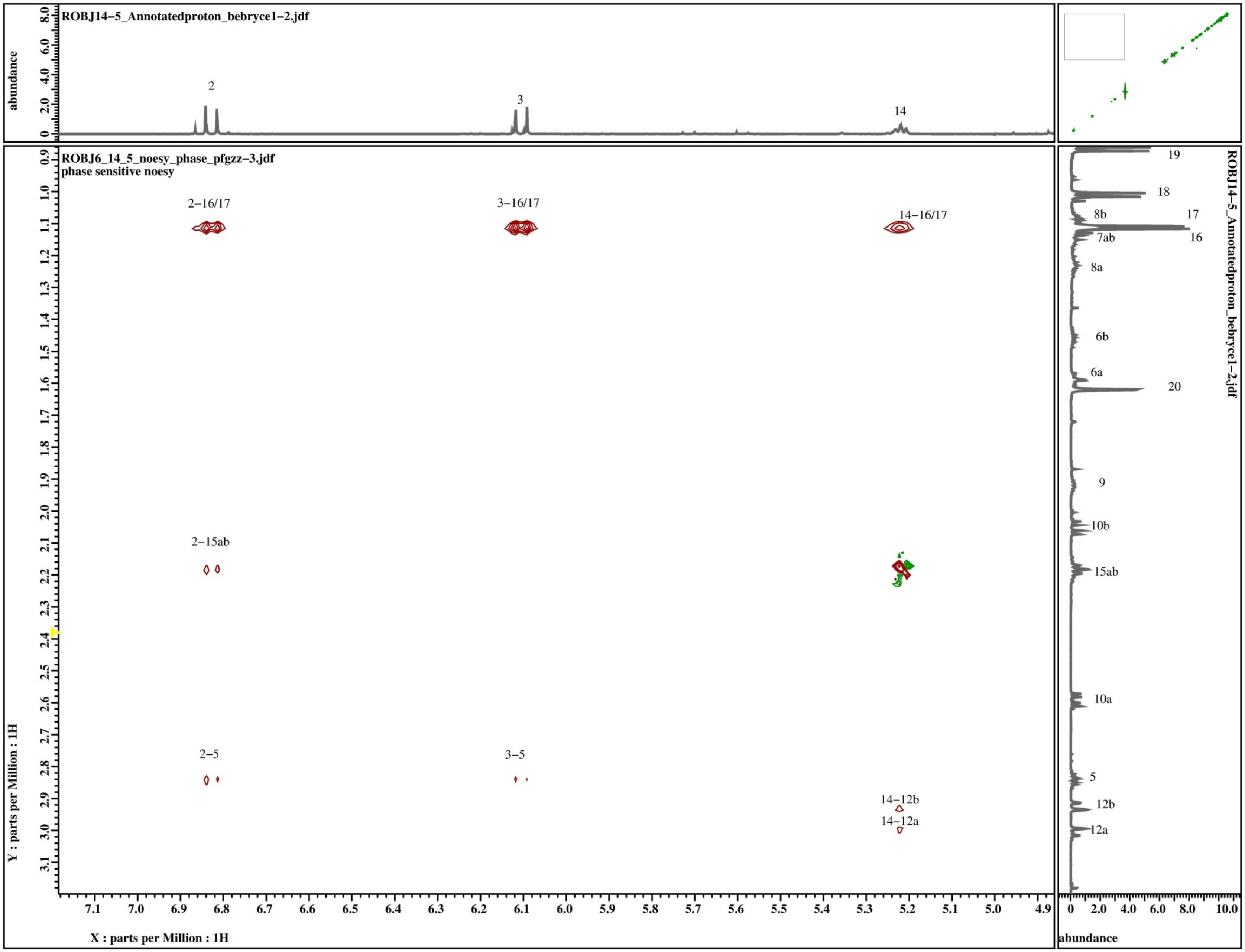

S18. 1D dpfgse-NOE spectrum of bebrycin A (600 MHz, methanol-d_4) irradiated at H-5.

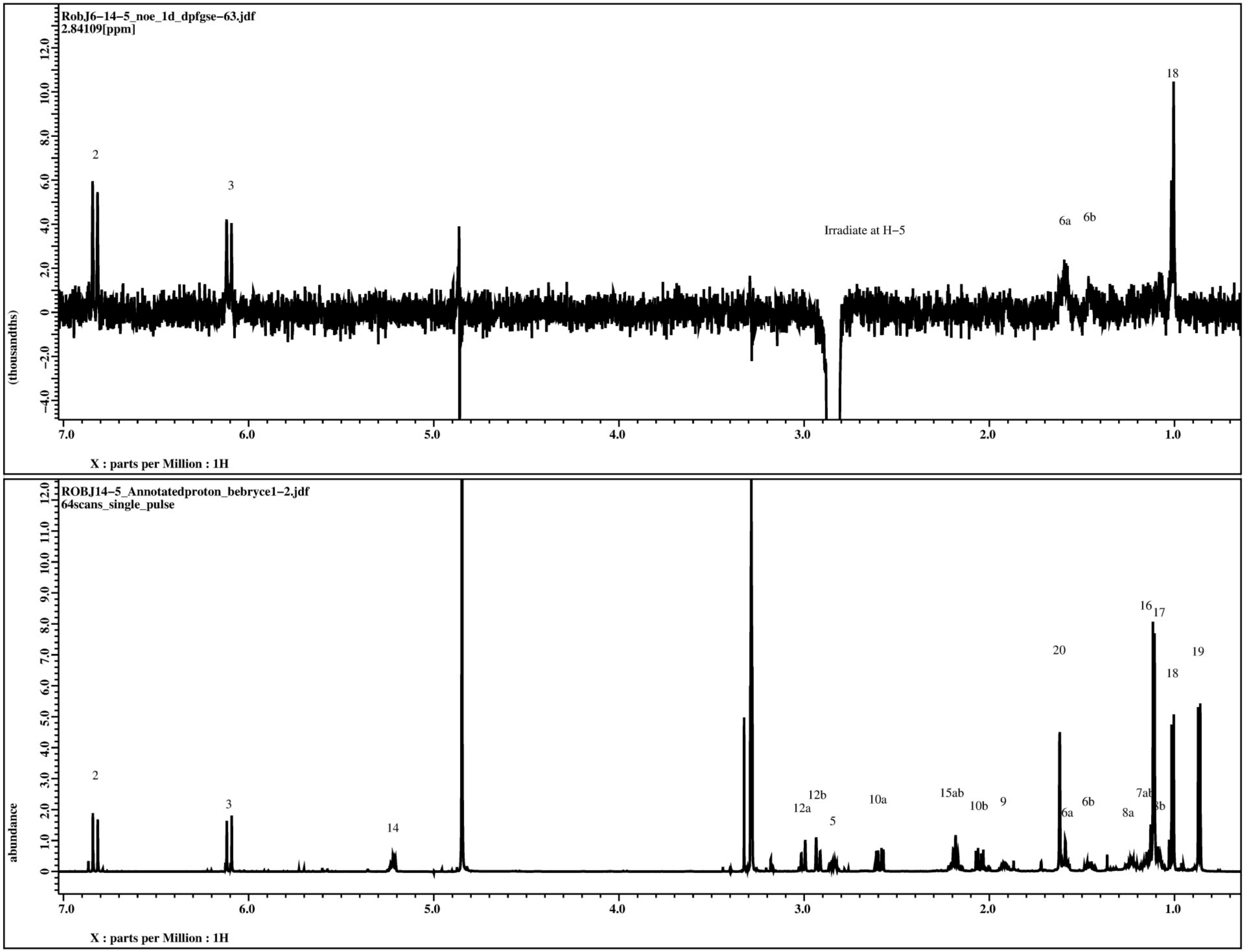

S19. 1D dpfgse NOE spectrum of bebrycin A (600 MHz, methanol-d_4) irradiated at H-18.

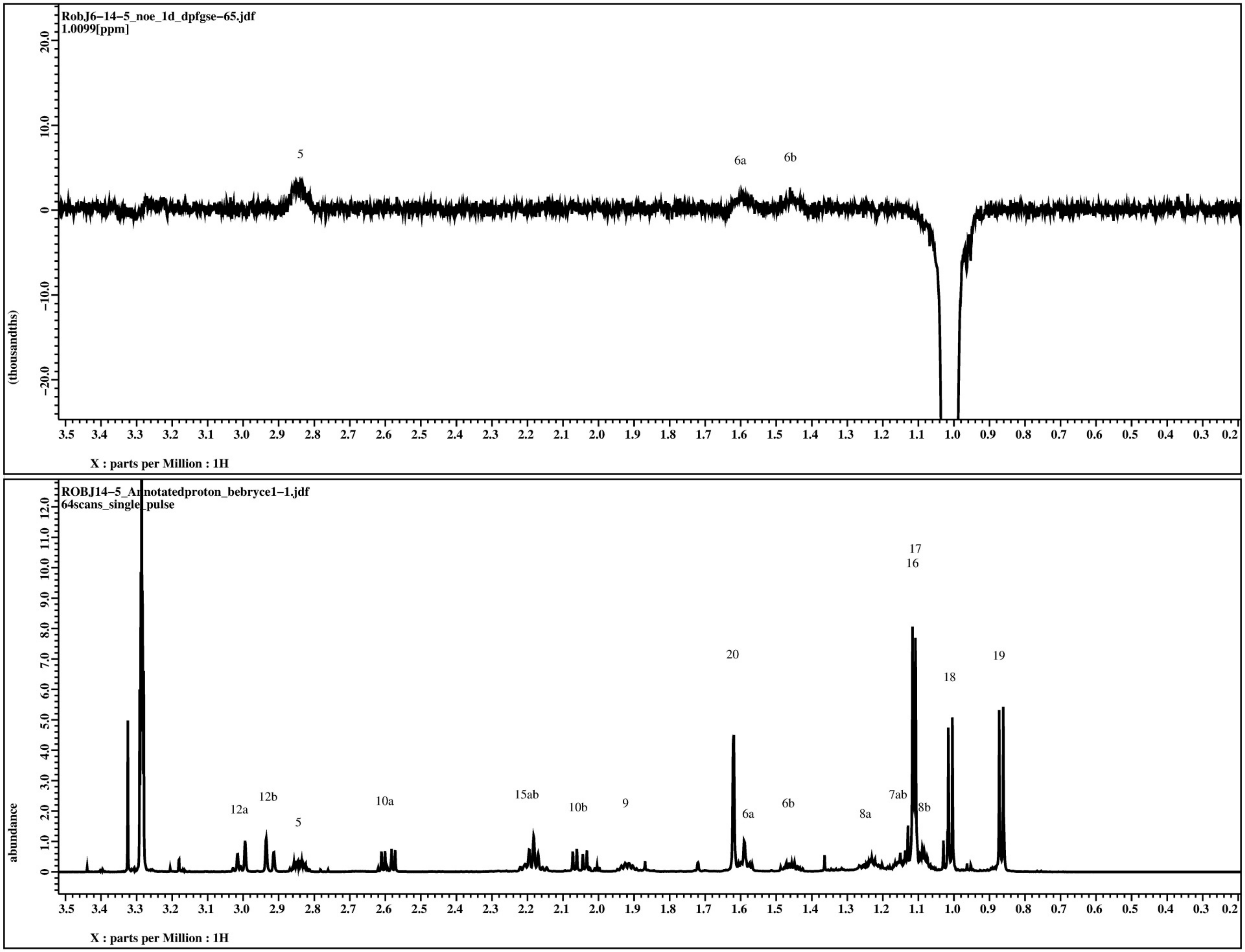

S20. 1D dpfgse NOE spectrum of bebrycin A (600 MHz, methanol-d_4) irradiated at H-14.

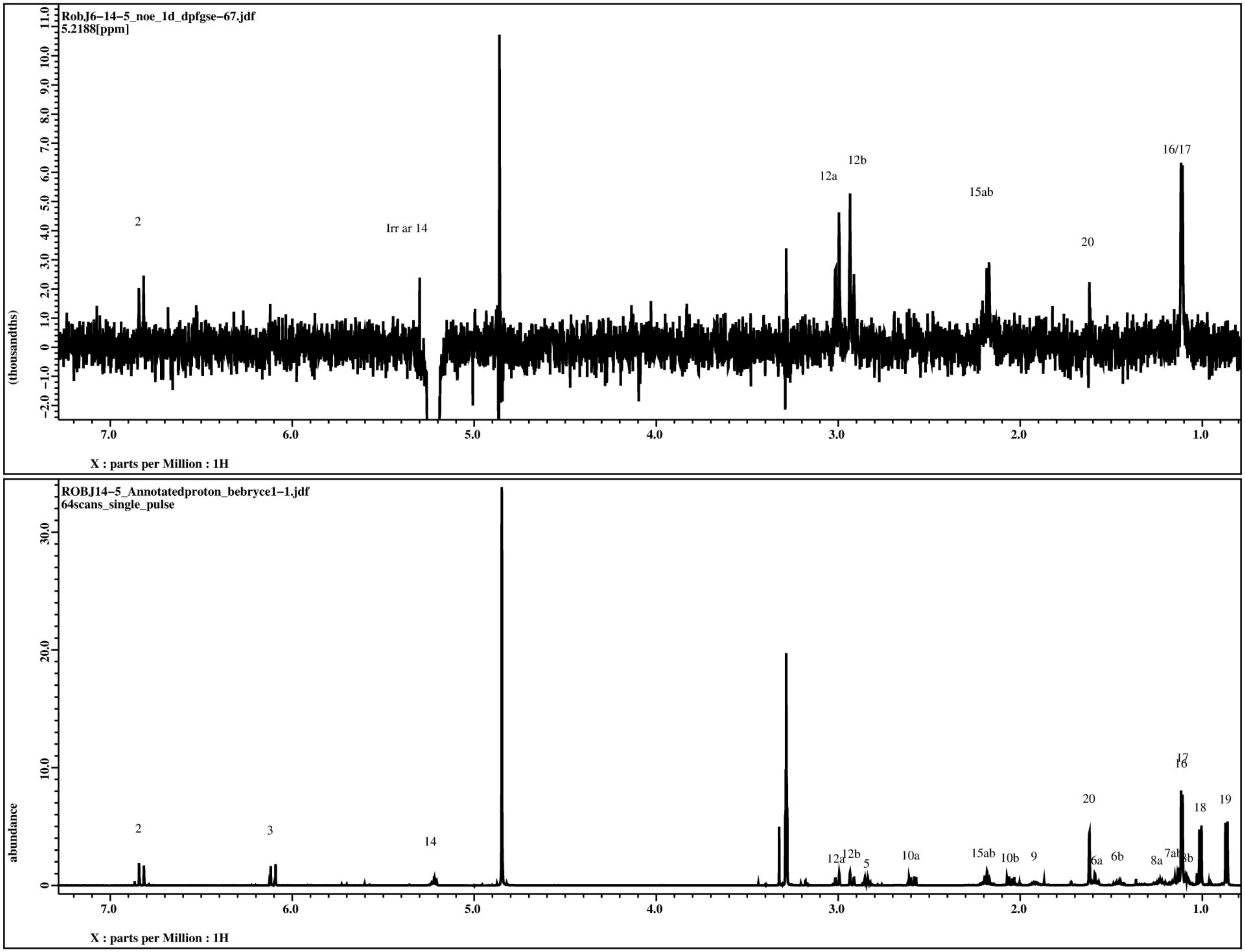

S21. 1D dpfgse NOE spectrum of bebrycin A (600 MHz, methanol-d_4) irradiated at H-2.

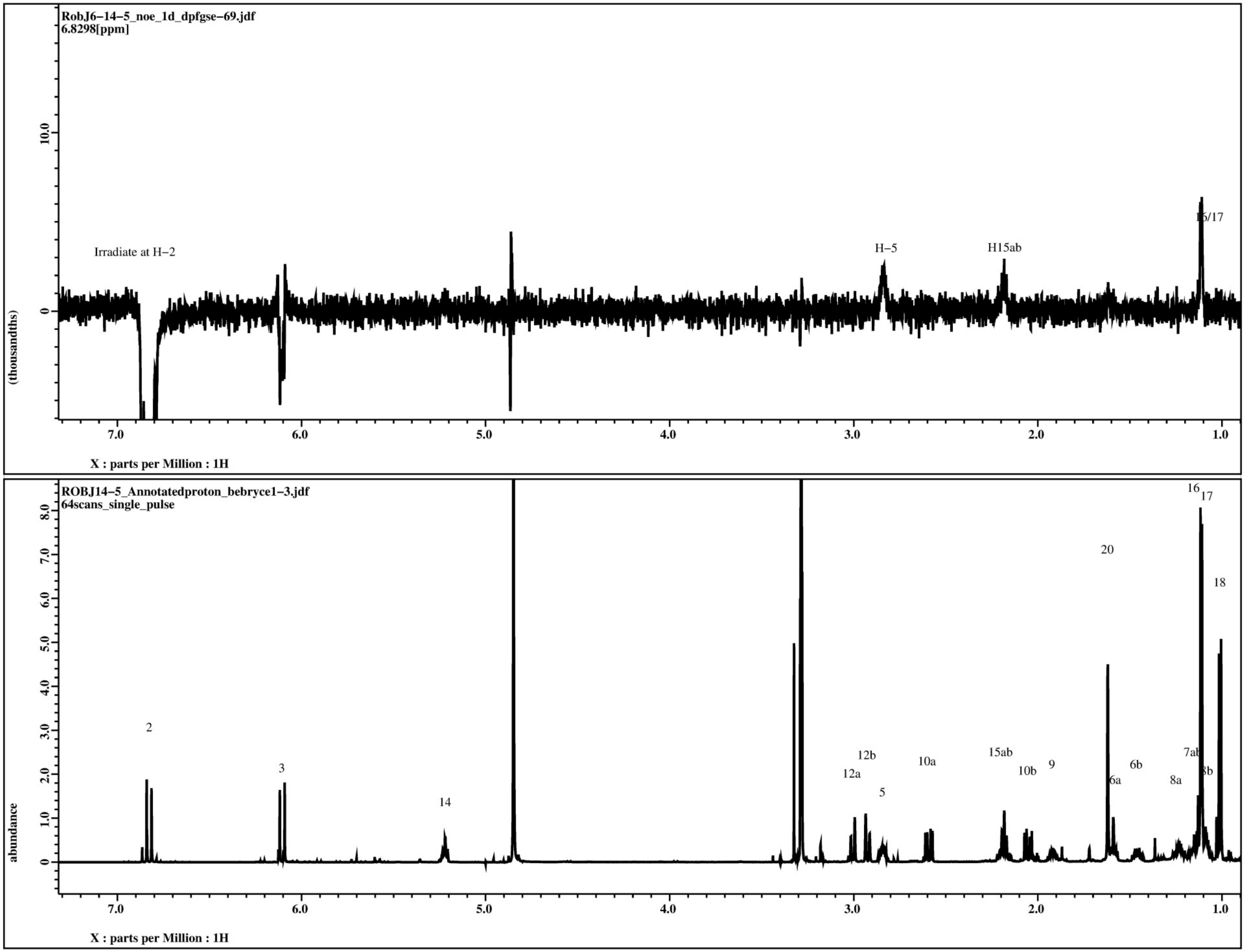

S22. 1D dpfgse-NOE spectrum of bebrycin A (600 MHz, methanol-d_4) irradiated at H-3.

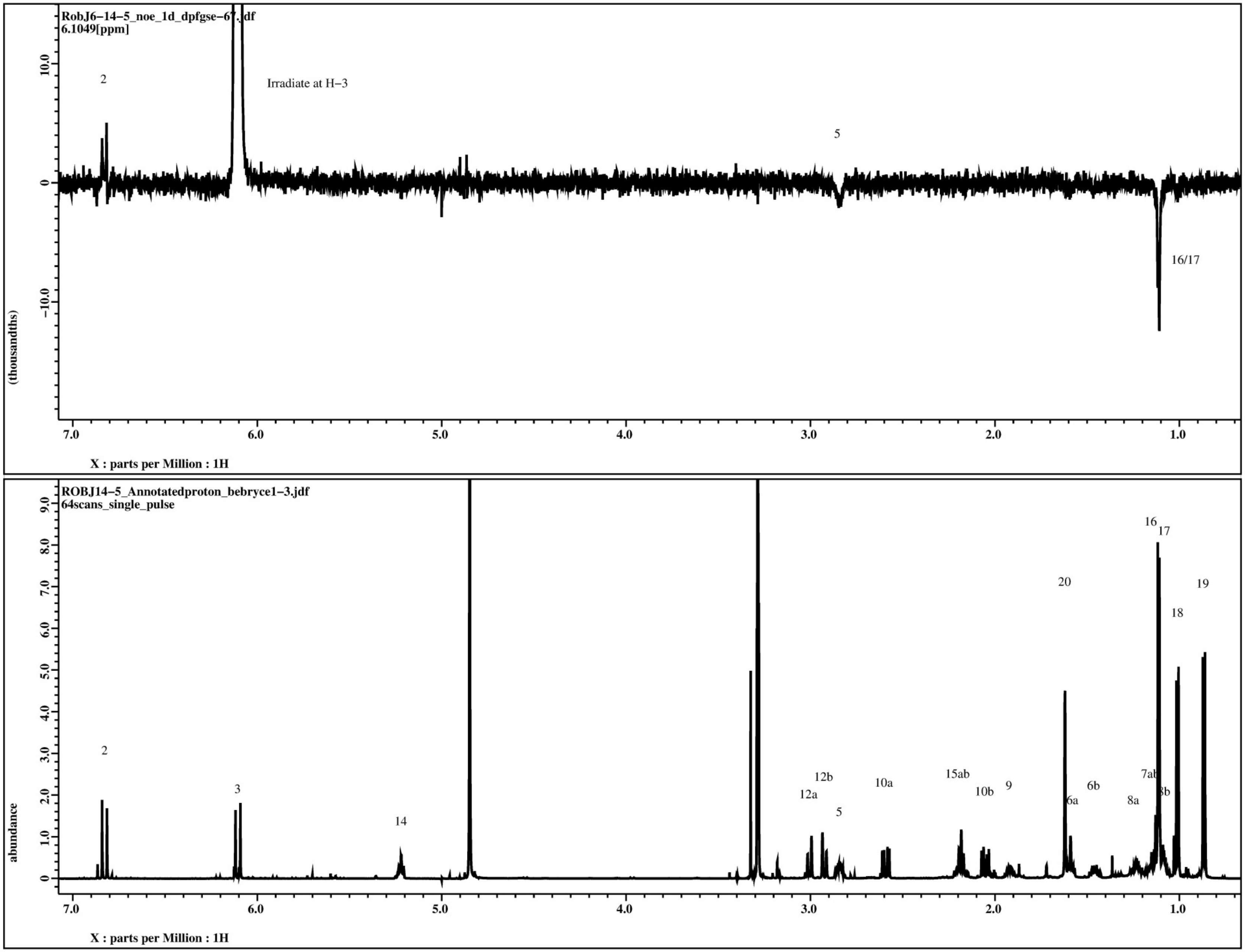

S23. HR ESI MS of bebrycin A measured on a JEOL AccuTOF Dart Instrument.

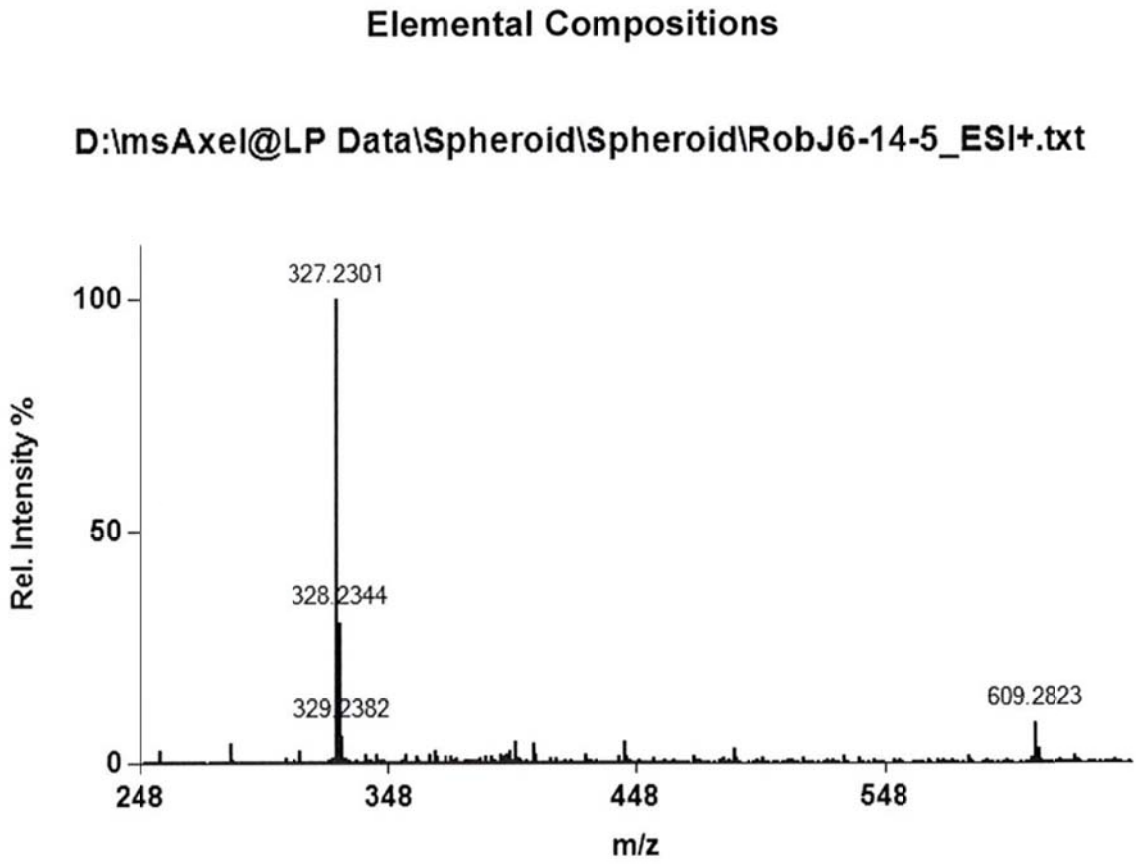

Mass Spectrum

Elemental Compositions
Element Limits: C 0/50 H 0/100 O 0/10 N 0/10 Na 0/1
Tolerance: 5 mmuEven or odd electron ion or both: Even
Electron correction: None.Charges: 1
Minimum unsaturation: -1Maximum unsaturation: 100

Calc. m/z	Abund %	mmu	DBE	Composition
327.234522	100.000	4.45	1.5	C10H28O1N10Na1
327.225695	100.000	-4.38	4.5	C13H27O2N8
327.225977	100.000	-4.10	0.5	C15H32O4N2Na1
327.227313	100.000	-2.76	5.5	C16H28N6Na1
327.228382	100.000	-1.69	3.5	C17H31O4N2
327.229718	100.000	-0.35	8.5	C18H27N6
327.230000	100.000	-0.07	4.5	C20H32O2Na1
327.232405	100.000	2.33	7.5	C22H31O2

S24. Biological material used in the isolation of nitenin (**2**).

The sponge (HBOI Sample ID: 5-VI-91-2-007) was collected in 1991, using the Johnson-Sea-Link I submersible, from a rock outcrop on a sand flat, 98.8 m deep, off the east coast of Fuerte Ventura, Canary Islands [Latitude: 28 09.57'N, Longitude: 14 06.10'W]. The morphology is lamellate to fan-shaped, with multiple lamellae arising from a common base. Individual lamellae are up to 15 cm wide, 20 cm high, and 5-10 mm thick. The surface of the sponge is microconulose and abundant oscula (1-2 mm wide, 5-10 mm apart) occur on one side of each lamella; on the other side are only ostia. The sponge was yellow-tan in color when alive. The consistency is dense and compressible. The skeleton is fibroreticulate, dominated by secondary clear fibers (20-30 μm in diameter), and less abundant primary fibers (50-100 μm in diameter) that are cored and covered by sand. Secondary fibers formed meshes 100-300 μm in diameter. The specimen is *Spongia lamella*, previously known only from the Mediterranean Sea, Atlantic coast of Portugal and the Straits of Gibraltar [Phylum: Porifera, Class Demospongiae Order: Dictyoceratida, Family Spongiidae, Genus/Species *Spongia lamella* (Schulze, 1879)]. A taxonomic reference sample is archived at FAU Harbor Branch Oceanographic Museum (HBOM), sample number: 003:00839.

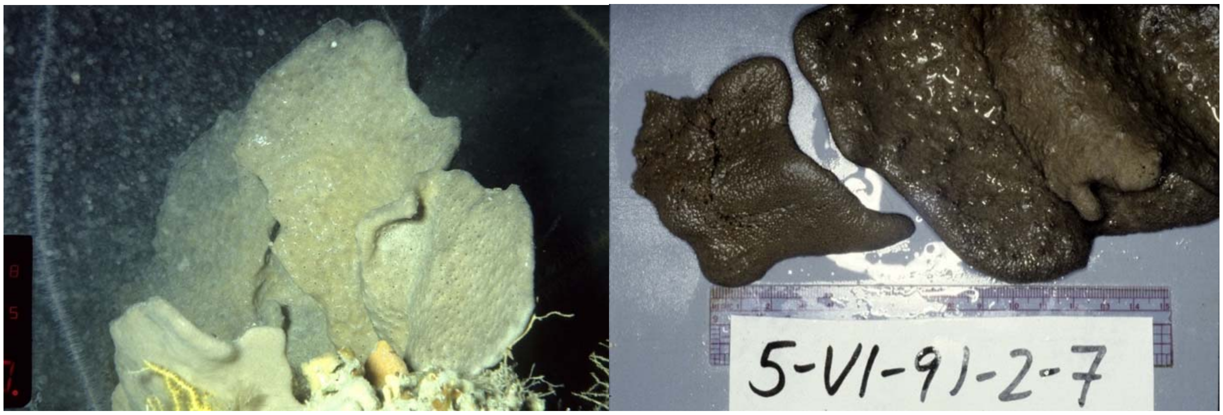

Figure 16A. Live specimen (left), deck photo showing two sides of lamellae (right).

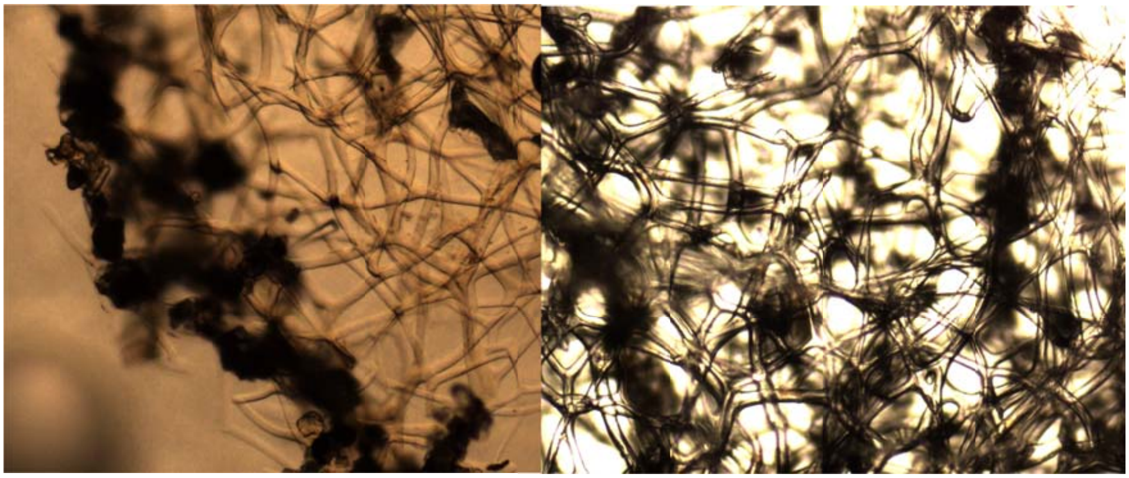

Figure S16B. Cross-section close to the surface with dermal sand layer (left); fibroreticulation of clear secondary fibers and rare primary fibers cored by sand (right). (4x magnification).

References for Sponge Taxonomy:

Pronzato, R., Manconi, R. (2008). Mediterranean commercial sponges: over 5000 years of natural history and cultural heritage. *Marine Ecology*. 29 (2): 146-166.

Schulze, F.E. (1879). Untersuchungen über den Bau und die Entwicklung der Spongien. Siebente Mittheilung. Die Familie der Spongidae. *Zeitschrift für wissenschaftliche Zoologie*. 32: 593-660.

Figure S25. Structure of nitenin (**2**) with numbering.

Structure of nitenin

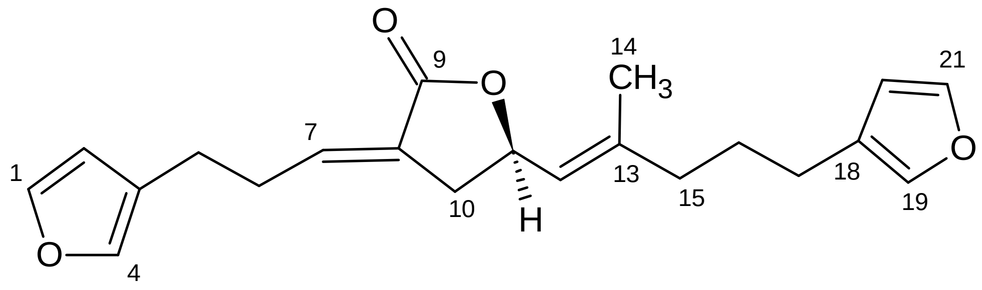

2

S26. Table of ^{1}H and ^{13}C data showing published data for nitenin in $CDCl_3$ and current isolation in CD_3OD, 600 MHz.

	Published CDCl₃		CD_3OD				
Position	δ^{1}H	δ^{13}C	δ^{1}H	δ^{13}C	COSY	HMBC[a]	NOESY
1	7.34, s	142.87	7.38, s[b]	144.23[b]	2, 4	2, 3, 4	2
2	6.3, s	110.93	6.32, s[c]	112.03[c]		1, 3, 4	1, 5
3		123.87		125.54			
4	7.24, s	139.06	7.29, s	140.46	5	1, 2, 3	5
5	2.57, m	24.23	2.57, t (7.6)	25.25	4	2 ,3, 4, 6, 7	
6	2.99, m	27.51	2.94, m	29.01	5, 7	3, 5, 7, 8	5, 7
7	6.16, dd (7.2)	142.07	6.24, tt (7.6, 2.1)	143.83	5, 6	5, 9, 10	5, 6, 10a
8	-	125.67	-	127.32	-	-	-
9	-	169.74	-	172.27	-	-	-
10a	2.99, m	36.46	3.08, m	37.37	10b, 11	7, 8, 9, 12	7,10b, 11/12
b	2.57, m	-	2.57, m	-	10a, 11	11	10a, 11/12
11	5.15, m	74.05	5.22, m[d]	76.2	10a, 10b	12, 13	-
12	5.22, d (8.8)	123.38	5.22, m[d]	124.87	14	14, 15	-
13	-	142.64	-	144.06	-	-	-
14	1.72, s	16.71	1.74, d (1.4)	16.74	-	12, 13, 15	11/12
15	2.06, t (7.6)	38.84	2.08, t (7.6)	40.01	16	12, 13, 14, 16, 17	11/12, 16, 17
16	1.68, s	27.69	1.69, m	29.15	15, 17	13, 15, 17, 18	15, 17
17	2.39, t (7.6)	24.23	2.40, t (7.6)	25.21	16	15, 16, 18, 19, 20	-
18	-	124.66	-	126.2	-	-	-
19	7.2, s	138.87	7.26, s	140.32	-	18, 20, 21	17
20	6.26, s	110.89	6.30, s[c]	112.01[c]	-	18, 19, 21	16, 17, 21
21	7.34, s	142.76	7.38, s[b]	144.13[b]	19, 20	18, 19, 20	20

[a]HMBC correlations, optimized for 8 Hz, are from proton(s) stated to the indicated carbon, [b,c,d]. Assignments may be interchanged.

S27. ^{1}H NMR spectrum of nitenin (**2**) (methanol-*d*$_4$, 600 MHz).

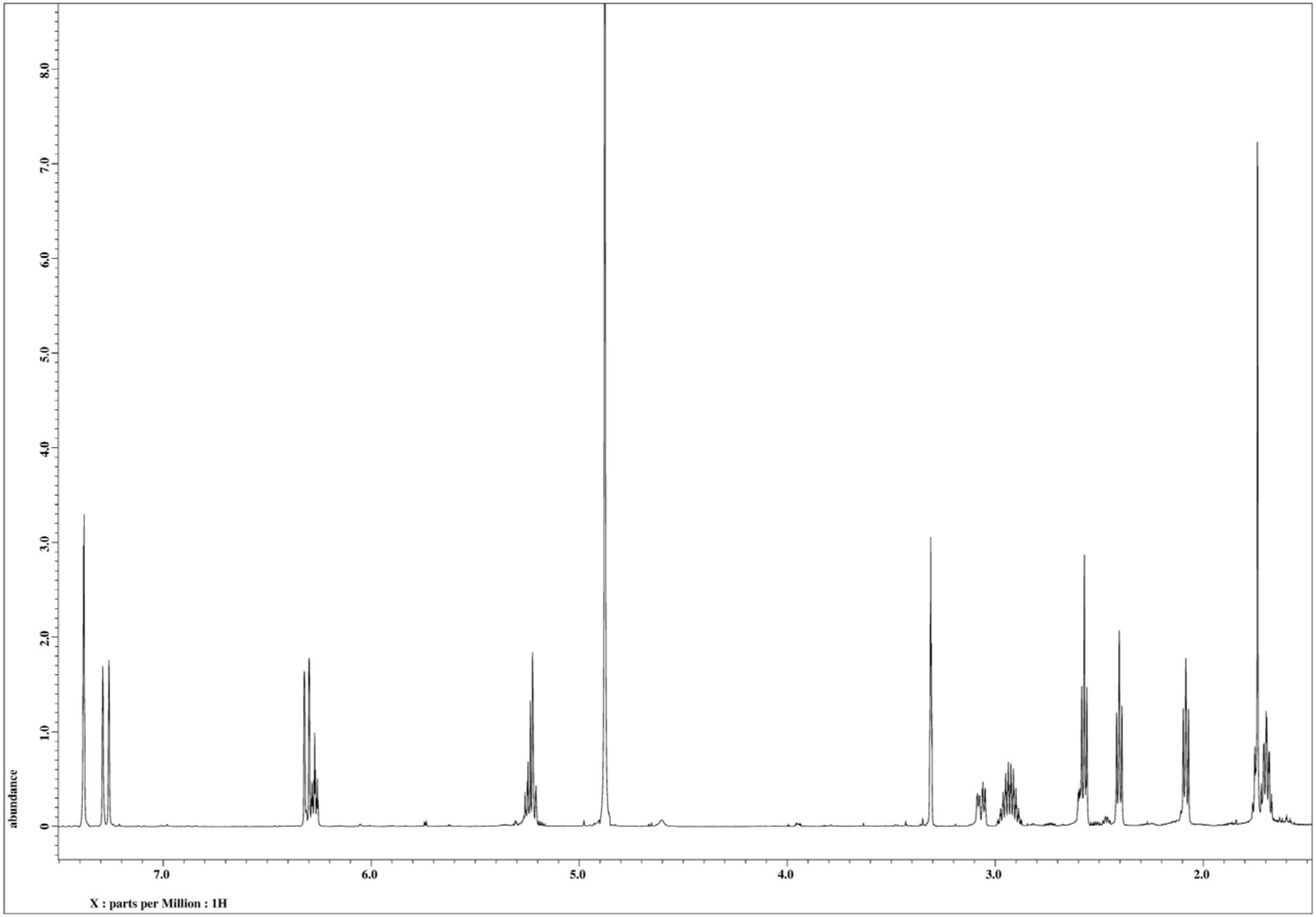

S28. Expansion of ^{1}H NMR of nitenin (methanol-*d*₄, 600 MHz).

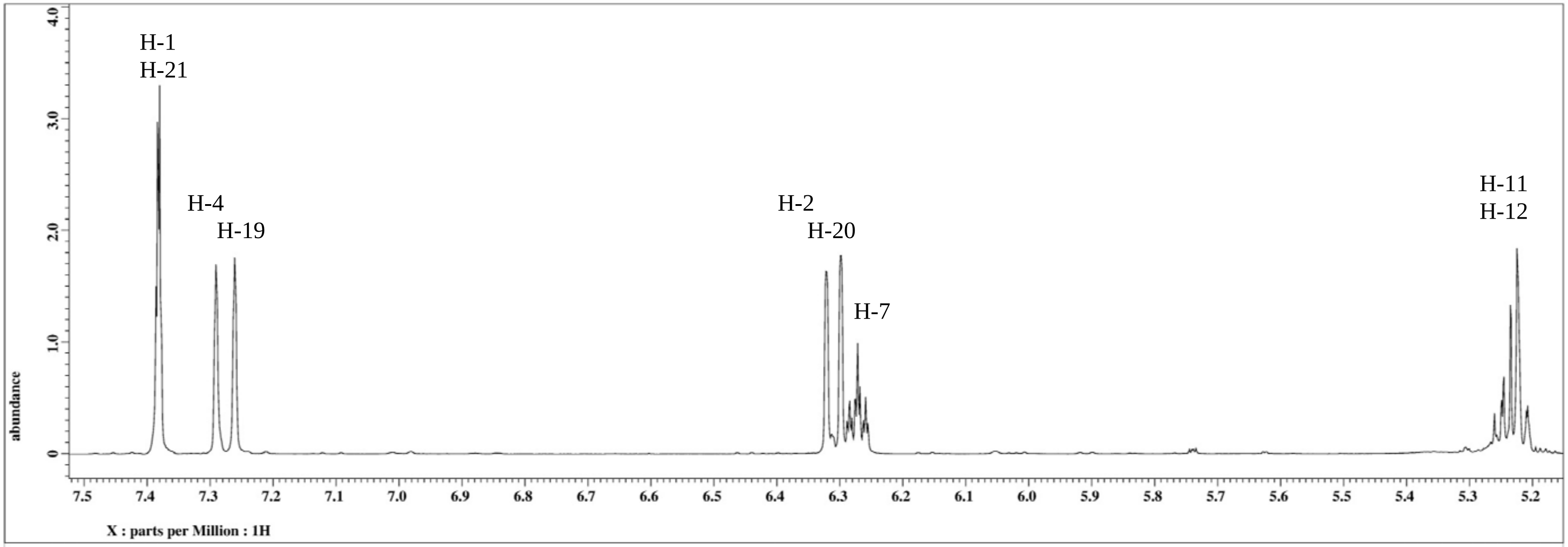

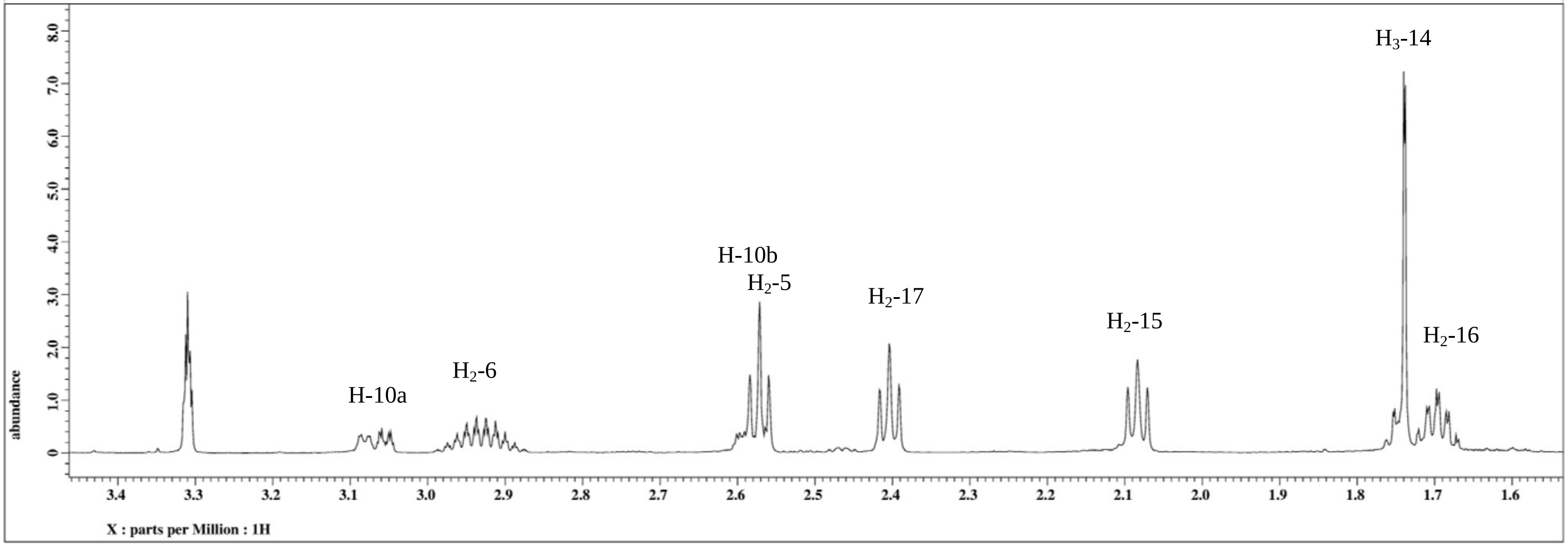

S29. ^{13}C spectrum of nitenin (methanol-d_4, 150 MHz).

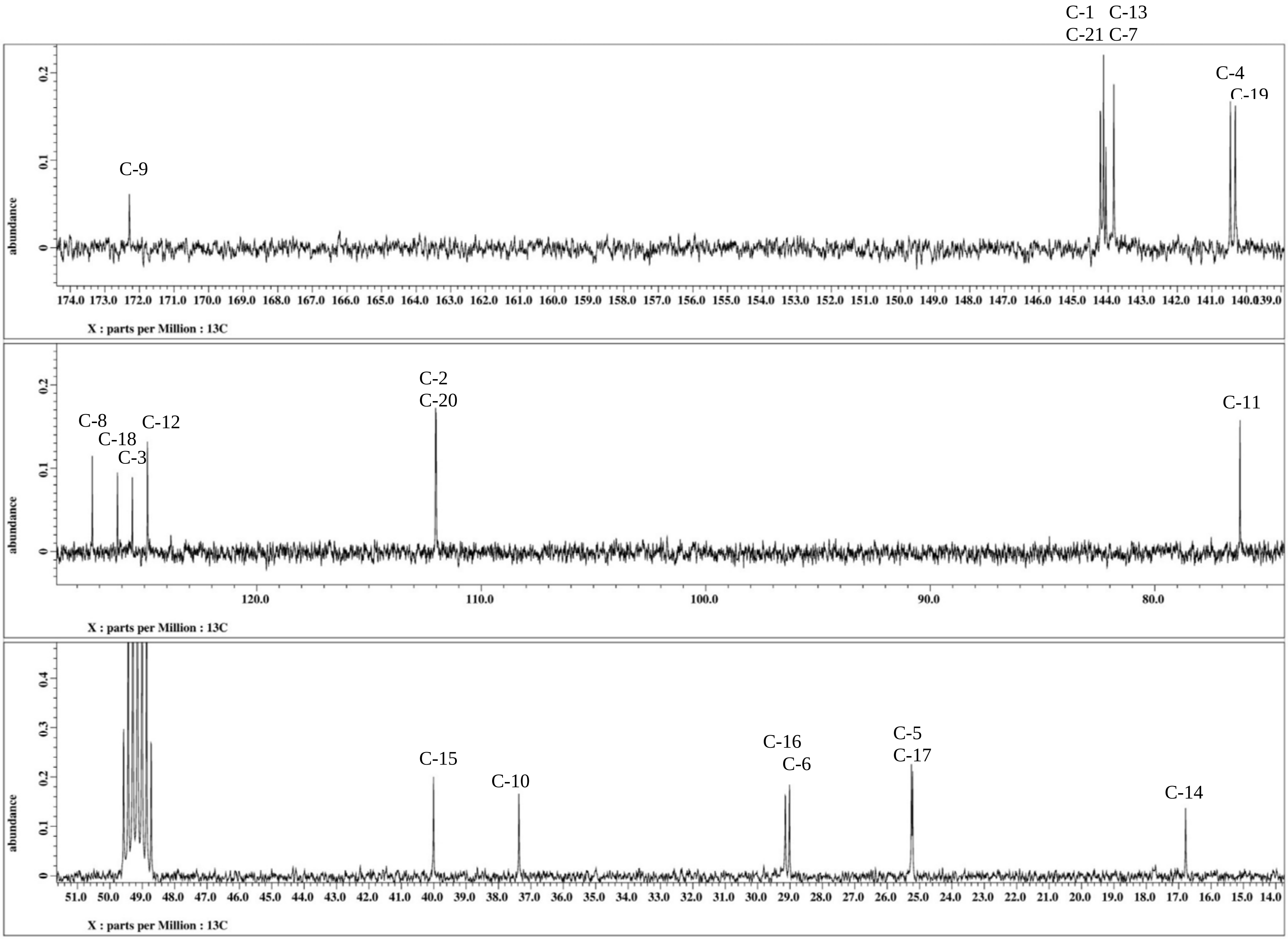

S30. 2D-DQF-COSY spectrum of nitenin (methanol-d_4, 600 MHz).

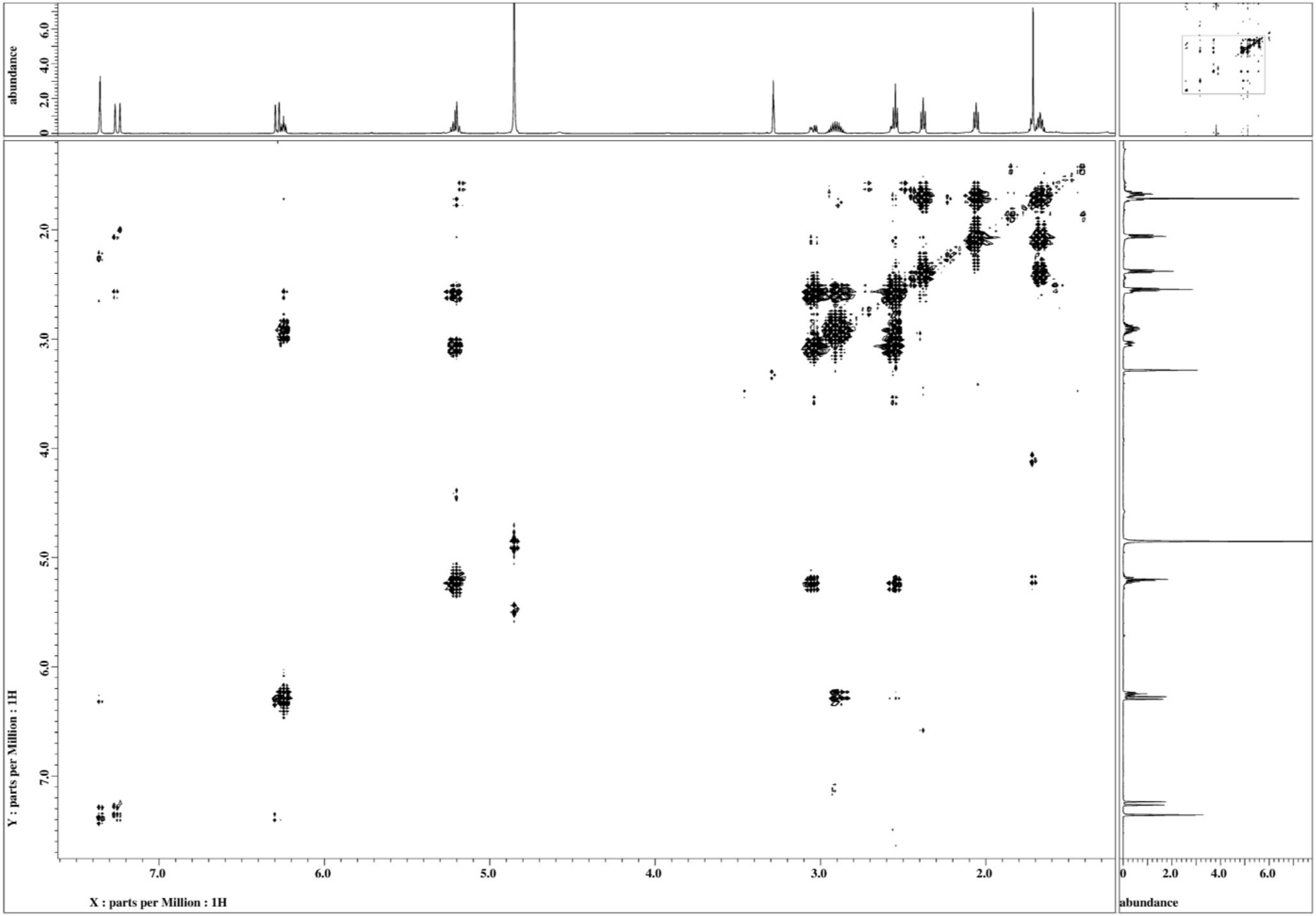

S31. 2D g-HMBC spectrum of nitenin (methanol-*d*$_4$, 150 MHz).

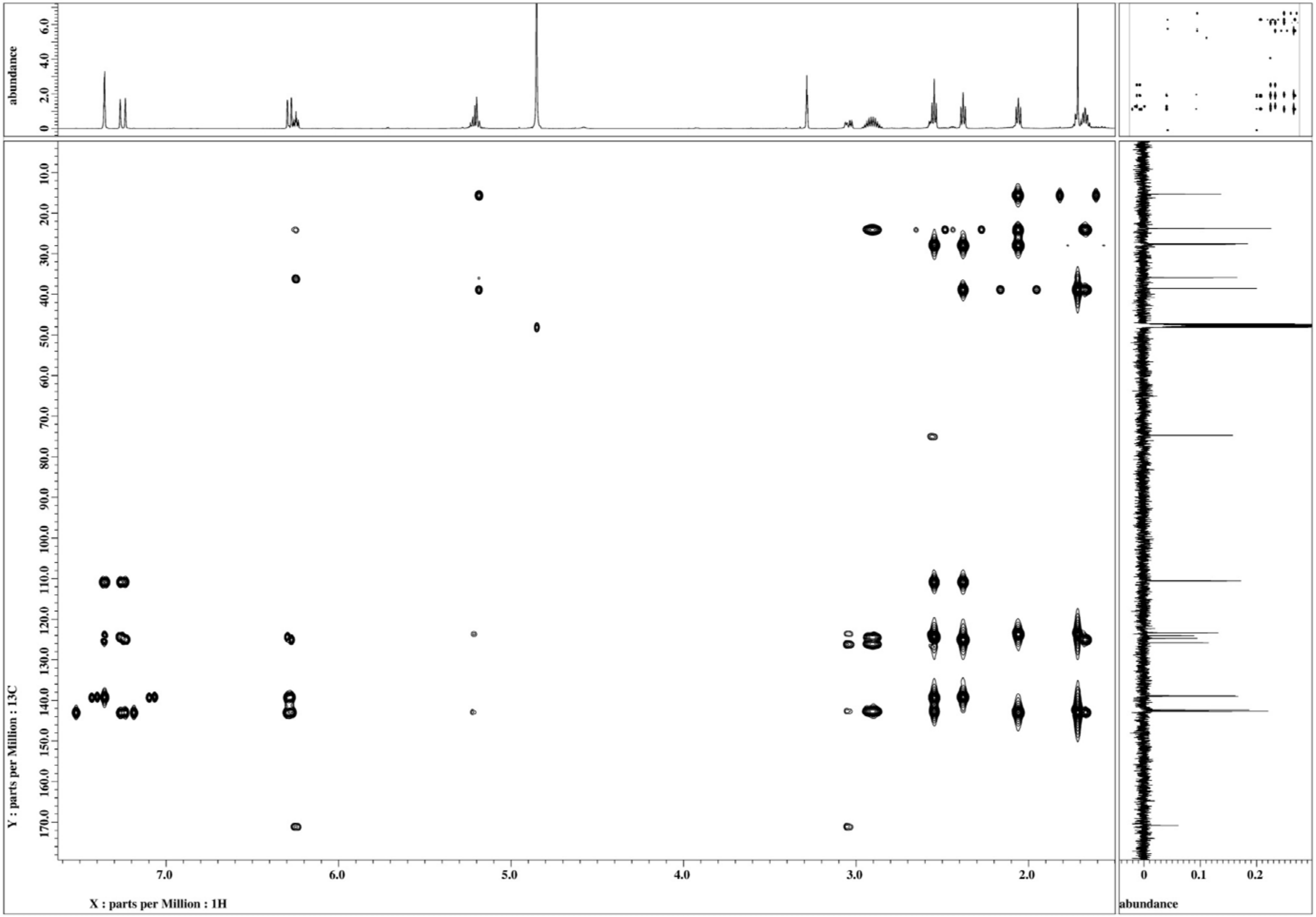

S32. 2D-edited gHSQC spectrum of nitenin (methanol-d_4, 150 MHz).

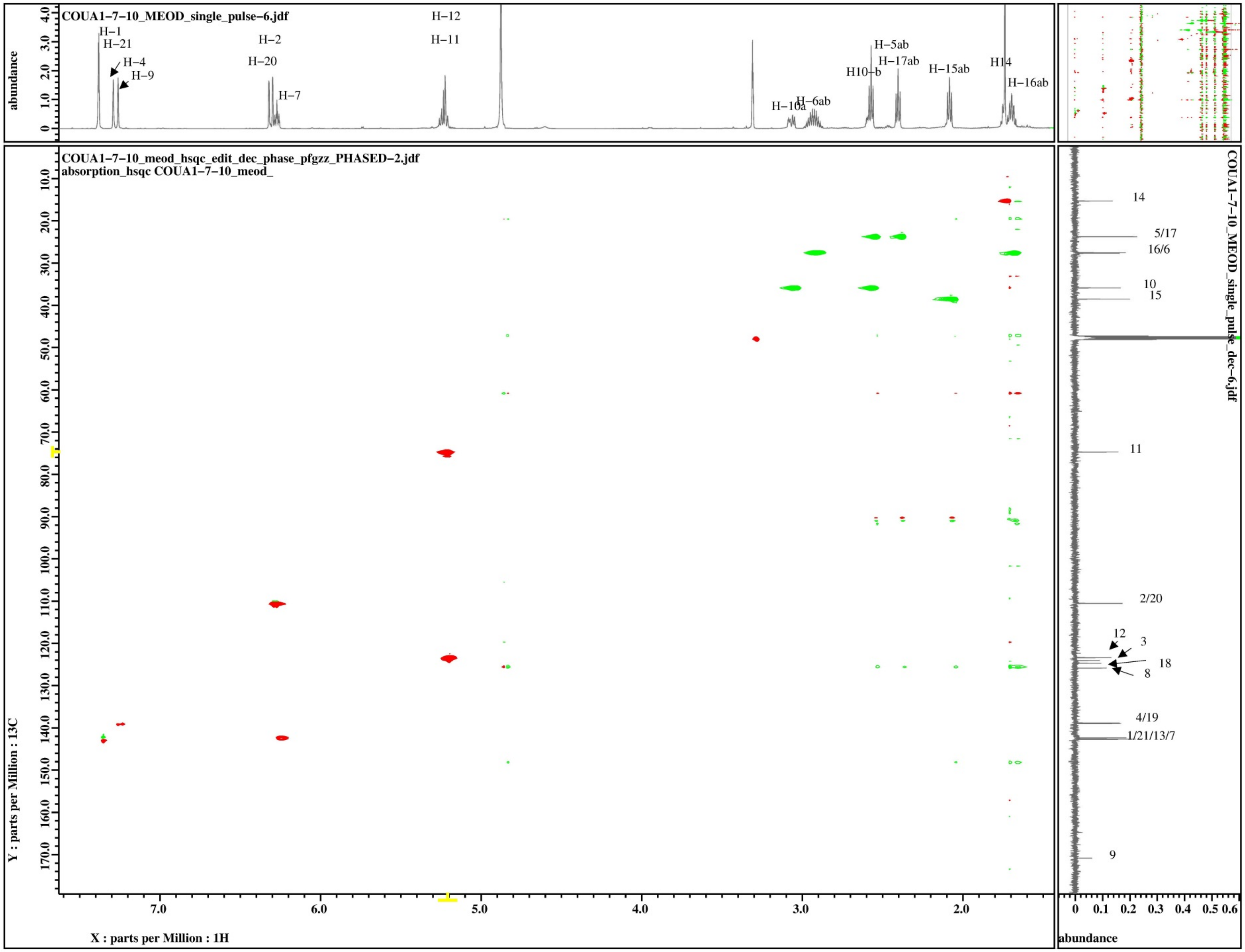

S33. 2D- NOESY spectrum of nitenin (methanol-d_4 600 MHz).

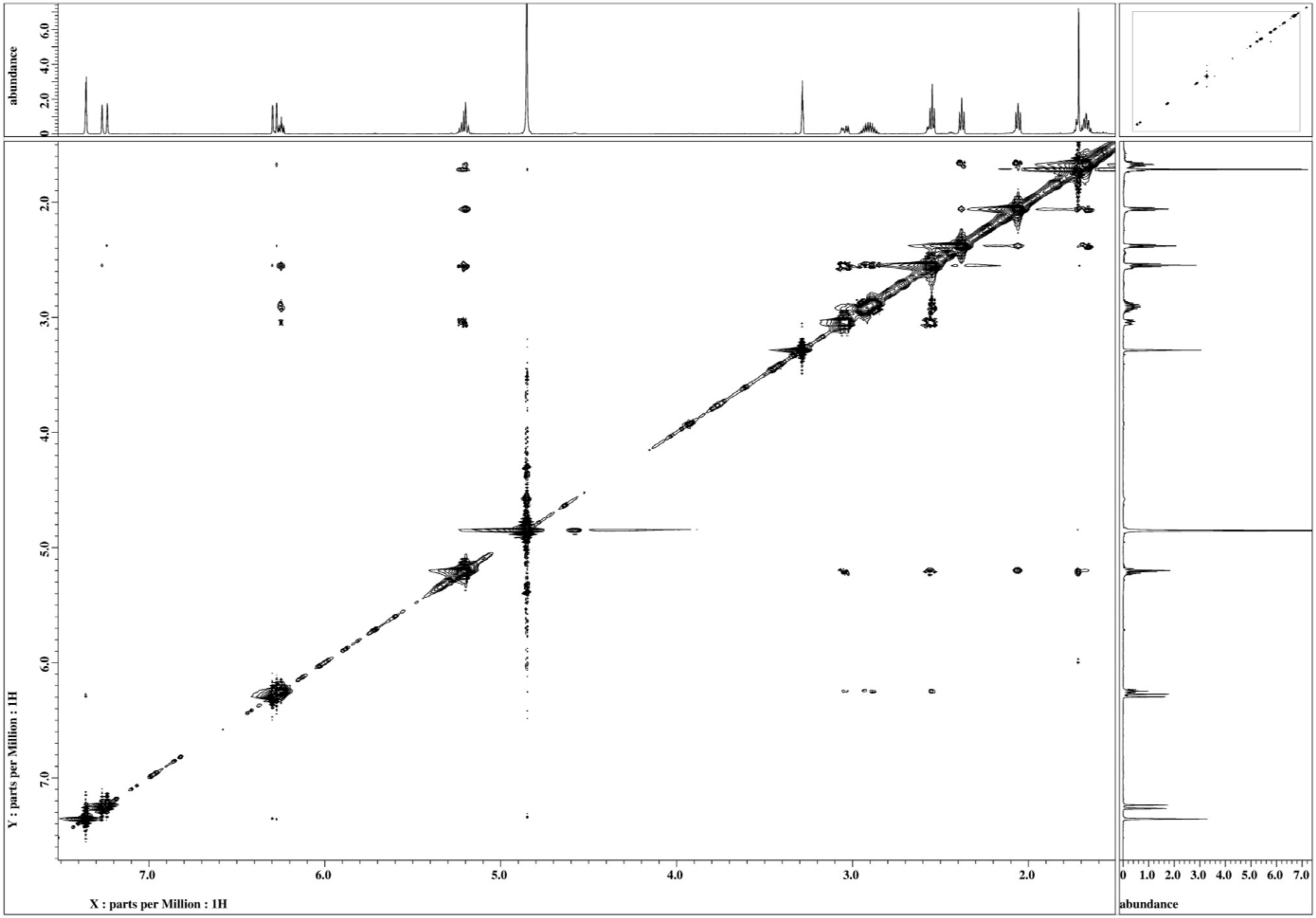

S34. HR ESI MS data (positive mode) for nitenin measured by direct infusion on a Thermo Fisher Orbitrap MS ESI ionization positive ion mode detection.

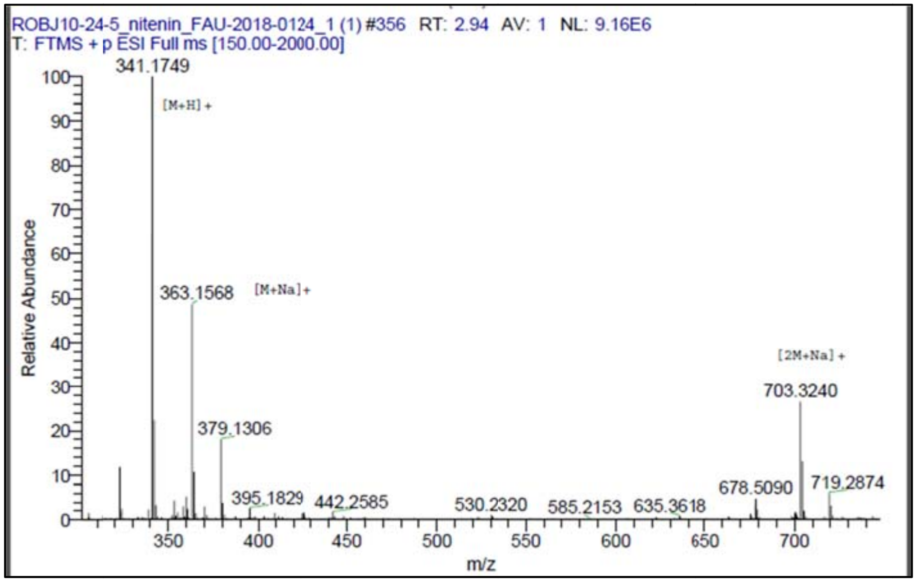

Observed: *m/z* 341.1749 $[M+H]^+$ $C_{21}H_{25}O_4$ Calculated: 341.1753 Δ=-0.4 mmu

Observed: *m/z* 363.1568 $[M+Na]^+$ $C_{21}H_{24}O_4Na$ Calculated: 363.1572 Δ=-0.4 mmu

S35. Stage specific interaction of nitenin (**2**) during *P. falciparum* intraerythrocytic maturation treated 30 hours post invasion (HPI). Synchronized Dd2 culture was exposed to the nitenin at 5 x EC50 at 30 hours post invasion (HPI) and monitored into the next life cycle stage up to 54 HPI. Untreated wells (containing DMSO vehicle) were included as controls (data for DHA is shown in the main paper Figure 3). Giemsa smears (inset) and flow cytometry with nucleic acid staining fluorophore YOYO-1 were collected every 12 h following compound addition at 30 HPI. Results represent the combination of three independent replicates.

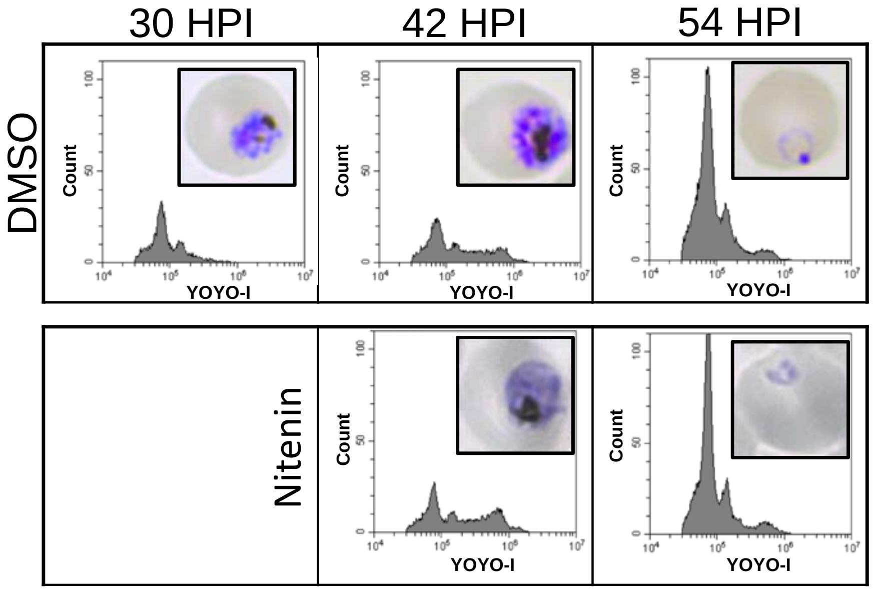

Supplement: Supplementary file 1 [file marinedrugs-19-00179-s001.pdf]
